# A multi-stage framework for emotionally intelligent dialogue systems using topological sentiment encoding and causal policy optimization

**DOI:** 10.1371/journal.pone.0361365

**Published:** 2026-10-28

**Authors:** Amreen Habibullah Khan, Amit Pimpalkar, Tabassum H. Khan, Sonali Mahure, Sireesha Koneru, Niraj Kumar Dewangan, Harika Vanam

**Affiliations:** 1 Computer Science & Engineering Department, MIT School of Computing, MIT Art, Design and Technology University, Pune, India; 2 School of Computer Science and Engineering, Ramdeobaba University, Nagpur, India; 3 Department of Artificial Intelligence, G H Raisoni College of Engineering and Management, Nagpur, Maharashtra, India; 4 Department of CSE, New Horizon College of Engineering, Bengaluru, Karnataka, India; 5 Department of Mechanical Engineering, Koneru Lakshmaiah Education Foundation, Vaddeswaram, Guntur, India; 6 Manipal Institute of Technology, Manipal Academy of Higher Education, Manipal, India; 7 Department of AI&ML, Gokaraju Lailavathi Engineering College, Hyderabad, India; Hunan Normal University, CHINA

## Abstract

Emotionally intelligent dialogue systems should not only express sentence-level sentiment but also sense shifts in emotion from sentence to sentence, from speaker to speaker, and over time, and be interpretable down to the decision level. Existing approaches consider only sentiment as a single and static feature, and they either do not consider multi-turn context, temporal variation, speaker-specific trends, or the correlations between emotion and action. To fill these gaps, we introduce a unified 5-stage framework for systematically encoding, transforming, and operationalising emotion in multi-turn conversations. First, Context-Aware Multi-Level Sentiment Encoding (CMLSE) uses RoBERTa embeddings, self-attention, and Dynamic Time Warping (DTW), while preserving the speaker identity, turn order and sentiment continuity. Second, Emotion Clustering Topological Signal Mapping (ECTSM) uses an emotion vector sentiment and persistent homology to obtain robust emotion clusters based on topology and shape. Third, Emotion-Memory Reinforced Graph Aggregation Network (EMR-GAN) constructs a dynamic emotion graph and a long-term memory network, learning the best manner for optimizing emotional coherence through Reinforcement Learning (RL). The Adaptive Causal Decision-Graph Transformer (ACDGT) incorporates causal inference into graph attention to discover hidden emotion–behaviour causality and imitate counterfactual decisions. Finally, the Emotion-Driven Reinforcement Policy Synthesizer (EDRPS) combines emotional memory and causal representations with dual-reward Proximal Policy Optimization (PPO) to generate policies in real-time, emotion-aware mode. Our model shows an average accuracy of 78.8% (+6.4% above the best baseline) on MELD, 0.61 Adjusted Rand Index (ARI) for emotion clustering, and 92.5% accuracy in aligning the policy on DailyDialog. These assessments show scores of empathy, relevance, and naturalness to be higher, marking a new state-of-the-art for affective dialogue systems.

## 1. Introduction

Emotionally intelligent dialogue systems must transcend traditional sentiment classification to understand the temporal evolution, causal triggers, and structural organization of affective states across multi-turn conversations. Dialogue systems need to be able to express naturalistic responses and to infer fine-grained sentiments and emotions that can be found in multi-turn dialogues, much like empathic humans in a contextual situation. Dialogue systems must advance beyond conventional Natural Language Processing (NLP) by encoding speaker identity and temporal order capabilities that standard sentence-level NLP does not provide while also striving to understand, adapt to, and generate emotional expressions. Despite significant advances in transformer-based architectures, current models [1] predominantly treat emotions as isolated utterance-level phenomena, failing to capture the dynamic interplay between speakers and the long-range affective dependencies that characterize real-world human interactions. Current models do not distinguish emotional triggers from incidental expressions. This limits their use in decision-making tasks that require causal understanding of emotion [2,3]. In a multiple-party conversation, speaker dynamics, turn-taking behaviour, and the flow of contextual emotion play a central role in creating the content of emotions, yet very little research has been done on these topics in conventional models. Traditional sentiment analysis techniques tend to make atomic-level evaluations at the sentence level, considering utterances either negative or positive [4]; however, these methods ignore the affective dynamics between conversational turns and between the speakers, which are best captured at the dialogue level. Traditional sentiment analysis methods usually make atomic evaluations at the sentence level, treating an utterance as positive or negative [5,6] rather than a dialogue level analysis that explains the evolution of the affective dynamics within and between conversational turns/speakers.

The graphical visualization as shown in Fig 1 illustrates the effect of emotions on dialogue and sentiment classification. The characters, Swati and Amit, make funny remarks about their felt emotions of surprise, joy, neutrality, anger, and sadness. A set of facial expressions is shown with each dialogue segment, and labels specify the emotion’s sentiment (positive, neutral, or negative). Existing models like BERT and RoBERTa present their own disadvantages: (1) They are based on fixed-length sequences, which causes them to miss out on capturing emotional relationships that are longer than the input sequence, (2) They cannot capture emotional connections that continue over time, and (3) They have no way to differentiate between emotional triggers and incidental emotional expressions [7,8]. Multi-party conversations in general are unique, in that their speaker dynamics, turn-taking behaviour, and contextual emotional flow heavily influence affective content [9,10].

**Fig 1:**
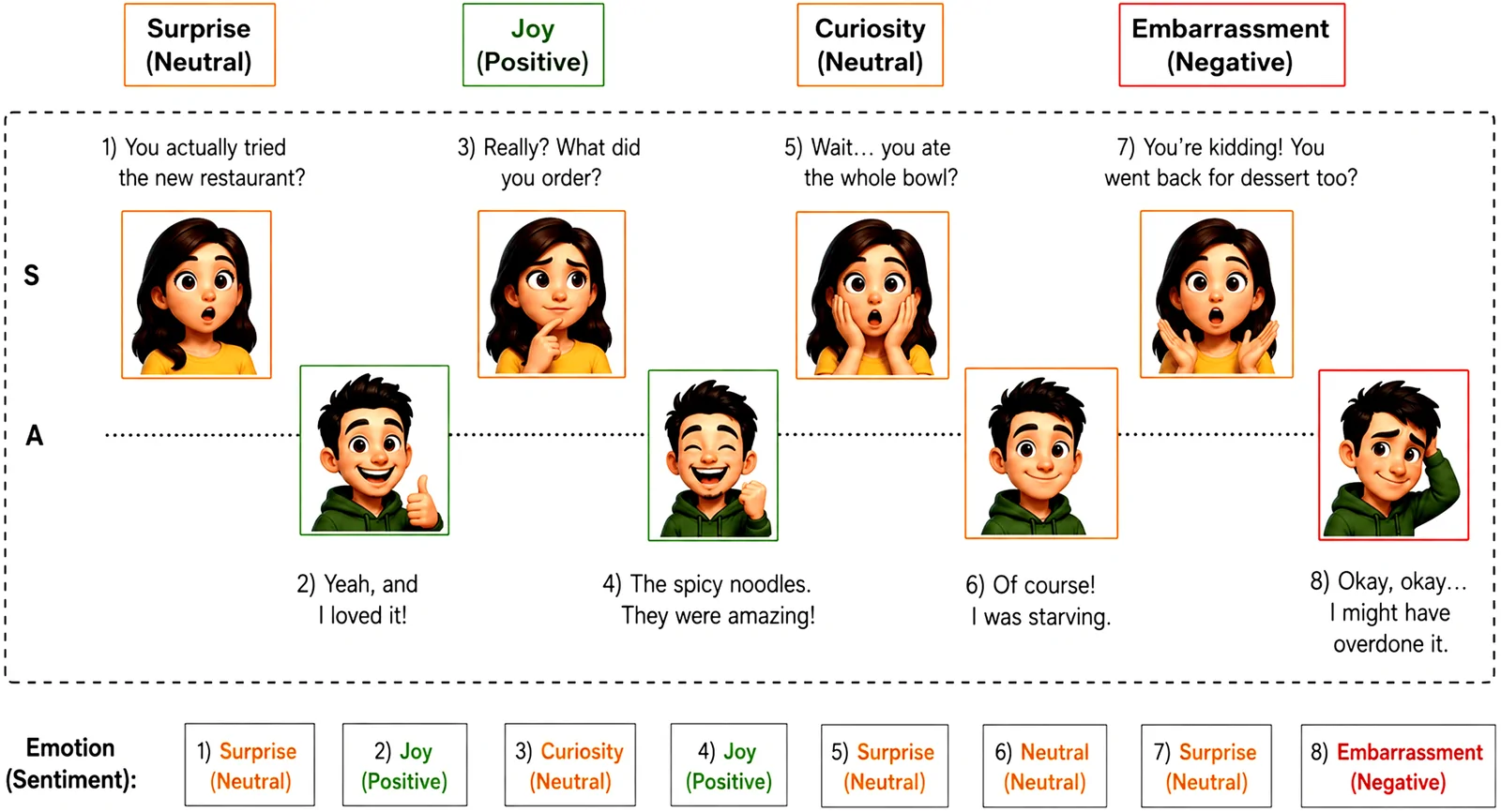
An example of emotion and sentiment in dialogue. Original illustration created by the author. The dialogue, emotion labels, and sentiment annotations are author-designed for illustrative purposes only.

### 1.1 Problem statement

In current dialogue systems, emotion is used as a one-shot, utterance-level label. Given a multi-turn conversation between two or more speakers, current models do not jointly address four specific problems. First, they fail to capture speaker identity and turn order in the sentiment, which means that they are unable to tell what emotion is expressed by whom at which turn. Second, they have no way of modelling the evolution of emotion over time across turns, so they cannot show how emotions relate between speakers. Third, they make no distinction between emotional cues and incidental emotional expressions, thus are unable to determine which speech caused which emotions later on. Fourth, they are not emotionally attached when it comes to downstream decisions and thus cannot provide a response that is consistent with the emotional thread of the conversation. In this work, these four problems are treated in a single framework. Formally, given a sequence of utterances $u^{\left(s,t\right)}$ produced by speakers s∈{1,…,S} at turns $t\in \left\{\text{1},\ldots ,T_{s}\right\}$, the objective is to produce an emotional representation for each utterance that preserves speaker identity, temporal order, and cross-speaker dynamics, and to use this representation to select a response $a_{t}$ that maximizes emotional consistency and causal fidelity with respect to the conversation history.

### 1.2 Motivation

The primary reason for this work is to cater to the growing demand for emotional intelligence in the system to navigate complex real-world scenarios. Systems in place often recognize emotions as a static, single-task behaviour, without considering changes in emotional triggers across conversational turns or those residing between speakers. This limitation prevents coherent emotional understanding in longer conversation dialogues-such as with therapy bots, social robotics, or empathetic virtual assistants. Additionally, this shortfall shows the notable disconnection in integrating emotional states into any further “downstream” decision making. While some works have included affective features in dialogue generation, the models that are available may not do that much with causal reasoning and end up producing emotionally inconsistent or context-insensitive responses. The current work proposes to improve these issues by proposing CMLSE as a dual-stage sentiment embedding that keeps track of the sentiments from different speakers over time using temporal attention and DTW. Nevertheless, a rare example of an approach to using topology in the field of emotion attaining worldwide classroom recognition, ECTSM presents a topologically-based clustering method that is very powerful against noise and outliers. Nevertheless, EMR-GAN is the modelling emotional memory reinforced using long-term affective coherence on a graph architecture. Following this, the EDRPS links understanding about emotion with policy synthesis, calling for RL rewards to act in line with emotional and causal constraints. Together, these motivate future steps where emotion representations become the input to downstream policy decisions and go beyond the traditional classificatory processes that aim to establish systems that also come to understand and decide emotions. The entire work makes a statement on emotion as a temporally dynamic, topologically structured, and causally influential signal that can evoke all meaningful feelings-something that lays the basis for true dialogue over emotions.

## 2. Related work

The current progress in emotion recognition has gradually advanced from unimodal, utterance level methods to multimodal, context-sensitive ones that utilize temporal relations and speaker interaction to build a more comprehensive emotional understanding. In their study, Yu and Chauhan [1] conducted an LDA analysis of NLP research to find the four main themes for personalized learning and also used sentiment analysis to assess the trends in NLP research. To bridge this gap, Yu et al. [2] introduced a dialogue emotion model that integrated local-global context encoding and multi-head commonsense knowledge attention for better emotion recognition in dialogues. The cognitive and emotional intelligence of GPT-3 was examined by Bojić et al. [3] via objective and self-assessment tests to determine if the human-like behavior and self-evaluations could be a sign of GPT-3 becoming subjective or conscious. Zhu et al. [4] conducted a study on the main technologies of emotion analysis in multimodal including text, speech, image and physiological signals, and analyzed the methods of feature extraction and early, late, model-based fusion methods.

The applications of AI in optimizing industrial systems by using language models were explored by Fu et al. [5] and Zhang et al. discussed about deep learning architectures for using language models for business-related chatbots for commercial NLP applications [6]. To ensure accountability of AI decision-making systems in critical sectors such as credit scoring, these ethical assessment systems were employed, such as those proposed by Pokholkova et al. [7]. Another thread focused on the affective aspects of education, such as motivation and burnout [8] as well as the epistemic boundaries in interaction with AI in a social-cognitive way [9]. Alyoubi [10] have continued this line of research, as they interpretably recognize multimodal emotions using a transformer-based model, thus introducing the aspects of technology transparency, trust, and usability. This was followed up by a study on fuzzy logic systems on emotional intelligence for hybrid work performance [11] that provided empirical evidence linking psychological traits such as self-esteem and creativity with translation technology competence [12]. The papers developed on multimodal recognition were a few ground-breaking ones in this line of work. Chauhan et al. integrated both audio and textual information to synthesize the context [13] and Zhu et al. presented a brain-inspired hierarchical perception model for emotion understanding [14]. The transition is now towards socio-cognitive integration, either as intelligent learning companions [15] or as AI literacy in the creation of innovations [16]. Nfissi et al. [17] demonstrated the effectiveness of convolutional-recurrent hybrids for speech emotion recognition directly from the waveform and Cao et al. [18] tackled empathetic dialogue generation by incorporating commonsense and emotion-cause. Emotion modelling for dialogue systems [19] and multimodal prompt learning architectures [20] were further developed in an expanding nuance space of how machines could behave when simulating and responding to human emotional expressions.

### 2.1. Emotion recognition in dialogue

Kátai et al. [21] explored the use of algorithms in education, showing that story engagement is associated with emotional involvement. The study by Arabian et al. [22] illustrated the applicability of emotion mimicry analysis to human–human interaction, underscoring the importance of empathetic mirroring in user engagement. Joseph and Maheswari [23] used deep learning techniques to measure facial expressions to detect emotions in smartphone addiction screening, connecting emotions with behavioral health. Layne et al. [24] focused on the topic of incivility in the process of professional socialization, thereby also implicitly focusing on emotional competence and control. Munawar et al. [25] established the impact of emotional intelligence on innovative behavior, mediated by psychological safety and servant leadership. Foote [26] gave a philosophical anthropology of humanistic management, emphasizing the emotional and relational aspects in work contexts. Clark [27] suggested that a way to lead positively is to be joyful, affirming the importance of the affective basis for effective leadership. Arda and Napoli [28] examined the attitudes of preservice teachers toward social–emotional learning by using children’s literature, further affirming the importance of emotional competence in learning.

Alhussein et al. [29] conducted a thorough meta-analysis to assess the accuracy trends and validate multimodal fusion over unimodal methods for the problem of speech emotion recognition in conversations. Bai et al. [30] proposed an EKBSA model by integrating a knowledge graph into the K-BERT model to tackle the Chinese sentiment analysis problem in conversational texts. Knob et al. [31] added empathetic AI assistants to everyday conversation by adding emotional intelligence modules. Tellai and Mao [32] introduced CCTG-NET that is a hybrid CNN-GRU network for SED using noisy datasets and achieved better accuracy. Lewandowski et al. [33] introduced multi-perspective evaluation approaches for conversational agent improvement that focus on continual improvement of the agents. Lal and Neduncheliyan [34] shared work about conversational AI in Healthcare and showcased the implementation of affective computing in healthcare. In their research, Akram et al. [35] studied the acceptance of recruitment chatbots and found that trust and ease of use are crucial for their adoption.

Emotion Recognition in Conversations (ERC) is becoming one of the core applications in affective computing, ranging from empathetic chatbots, mental health monitoring, to social robotics [36]. EF-ERC unified emotional flow modelling and contextual semantics, and employed closed-form, continuous-depth networks to capture the complex temporal relationships among emotional flow. Text-based emotion recognition approaches have been fairly successful, but emotion perception requires a greater sense of emotion than any one modality can provide in real-world dialogue systems. The study by ChengYan et al. [37] did a systematic analyze of multimodal emotion recognition in conversations that included core tasks, representative methods and evaluation strategies. Singh et al. [38] took this line further by improving conversational AI with emotion recognition techniques that rely on multiple modalities. However, current ERC approaches mostly depend on the global representations of context and/or local dependency modeling, which are not sufficient to reflect the subtle expression of emotions in multi-party conversations. Traditional approaches only consider individual positive/negative examples instead of considering emotional evolution throughout the turns in a conversation, which highlights the need for dialogue-level emotion modeling, considering the interaction of the speakers and the flow of the conversation. As a group, these works demonstrate the variety of emotion recognition forms, including facial expression, mimicry, organizational, and educational settings, indicating the wide range of applications that can be explored by dialogue systems capable of interpreting and responding to human affective states in complex contexts.

### 2.2 Temporal modeling of affect

The reviewed literature has very limited knowledge of temporal modeling of the evolution of emotion in dialogues. Zhang et al. [39] examined the persuasive factors and arousal levels of viewers for virtual AI streamers and their interaction with livestream shopping, finding a gradual pattern in emotional responses and purchasing over time during a livestream session. They discovered that arousal shares in determining persuasive effects; that is, arousal is variable over time and it affects consumer behavior. Cakmak et al. [40] investigated algorithmic drift in YouTube’s recommendation system and the consequences of longer-term changes in content exposure and user preferences, including polarization and bias. Temporal evolution of emotions in dialogue poses a basic issue, because a speaker’s current emotional state could be affected by the reactions of others in the dialogue and his/her own prior emotional states [41]. Wang et al. [42] presented a temporal-aware attention network to capture the temporal structures in emotion recognition, which is a very explicit approach considering the sequential relationship between utterances. Yuting et al. [43] proposed a dynamic causal disentanglement model with hidden variables to model the time accumulation of emotions. To better model the temporal aspect of node representations, Jingdie et al. [44] proposed ETCE-Net, which incorporated a temporal encoding module, which explicitly used a cosine-based mapping function to model sequential utterances’ dependency, thereby improving the temporal awareness of node representations. While these developments have encouraged a new way of thinking, emotions remain mostly given an episodic nature and nonlinear contextual interactions remain underrepresented in existing approaches, even when processed as discrete labels. Although neither study explicitly models temporal emotion evolution at a dialogue level, they emphasize the importance of temporal progression in the development of affective states across a consumer’s experience or in the experience of content consumption. The insights further highlight the need for dialogue systems to track the emotional trajectory over dialogue turns since the emotional states of the user are dynamic and change over time, depending on the flow of the dialogue, and changing contextual cues.

### 2.3 Causal reasoning in dialogue systems

Causal reasoning in dialogue systems has not been extensively discussed in the surveyed literature. Emotional triggers in speech have become more popular for distinguishing true emotions from incidental ones because of causal reasoning. By analyzing teacher activity mediation in data team meetings in the context of neo-liberal education reform, Rusnak [45] was able to uncover causal relationships among professional competence, teacher activity, and student outcomes. Elrawy and Wagdy [46] examined Egyptian architects’ perceptions of generative AI, revealing the factors leading to AI adoption and shaping the architects’ professional identity. Their analysis highlights a causal dimension to the attitudes towards AI, which is also relevant to the causal effects on user emotions in the context of dialogue agents. Hao et al. [47] introduced a causal emotion entailment model based on emotion-cause hybrid modeling to facilitate ERC by designing a bidirectional reasoning network to extract emotional information and simulate the complex emotional cognitive behavior of humans. Emotion-Cause Conversation Dataset (ECC) is the first automatically generated synergistic dialogue dataset proposed by Yuanyuan He [48] that simultaneously generates dialogue and emotion-cause labels. The CDEA framework [49] took advantage of LLMs to accomplish causality-disoriented conversation feeling analysis and focused on the causal effect of the earlier sentences from multiple speakers on targeted emotion. In a two-tier hierarchical causal model, HE-CGM proposed by Jianbang et al. [50], the model selects two VCAs to separate discrete emotion categories from continuous intensity levels with F1 scores of 79.83 and 96.31, respectively. Zhang Y. et al [51] implemented a sliding window multimodal feature fusion CauseMotion method to fuse emotional causal dynamic mapping with the multimodal approach, which improved the causal accuracy by 8.7% compared with other approaches.

However, the current causal models are still limited in their ability to distinguish emotional triggers from incidental expressions, thus restricting the use of the model in decision making tasks that involve the understanding of emotional causality. These limited causal reasoning interactions highlight the need to explicitly use causal modeling in dialogue systems. Although they do not directly address dialogue emotion causation, they illustrate causal reasoning methodologies which can be adapted to model causal chains in conversational affect: mediation, pathway analysis, and factor attribution. These methods offer a roadmap for building dialogue systems that exceed the scope of correlational patterns to deduce real-world emotional cause and effect, allowing them to respond more coherently and appropriately within the context of the conversation.

### 2.4 RL for dialogue policy

However, there were still few cases of dialogue policy optimization by RL reported in the literature. RL has proven to be an effective paradigm for optimizing dialogue policies with respect to emotional dynamics. Li et al. [52] studied the influence of scripted versus non-scripted collaboration in an online learning environment using a conversational agent. They have shown that adaptive scripts that modify according to the learner’s interaction have much greater impact on engagement and participation than non-adaptive scripts. Yue et al. [53] developed a dialogue world model that predicts the next speech and the user’s beliefs about emotion, sentiment, and intention with state-of-the-art accuracy in emotion classification, using model-based RL. Patrick and Chung [54] proposed a PPO-based conversational model that is capable of adapting to the emotional states of users in multi-turn dialogues, reconfirming that adaptive models over time can enhance user engagement and decrease the stress level of users. Wang et al. [55] proposed a verifiable emotion reward objective for character-coherent emotion-aware role-playing dialogue. The MindChat-R0 framework [56] introduces emotion reasoning as a method for RL to enhance the emotional support conversation system. Demotte et al. [57] suggested the capsule network architecture for sentiment analysis from social media, which comes with the introduction of hierarchical word-level relationships via dynamically routing capsules. They achieved 86.87% accuracy on Twitter Sentiment Gold and 82.04% accuracy on the CrowdFlower US Airline datasets without the help of handcrafted linguistic features. These methods have some limitations that are closely related to multi-turn dialogue: (1) they focus on individual sentences or small text chunks without modeling speaker-specific dynamics or turn-taking behavior; (2) they assign a sentiment label to a complete sentence, which means that they do not capture the temporal evolution of sentiment across conversational turns; (3) they do not differentiate between emotional triggers and incidental emotional expressions; and (4) they do not link representations of sentiment with downstream decision making or policy creation. Our work presents a multi-stage framework for sentiment encoding that utilizes speaker identity, temporal alignment, topological structure, long-term memory, causal relationships, and policy synthesis. The proposed multi-stage framework, which extends the work of sentiment encoding, aims to include speaker identity, temporal alignment, topological structure, long-term memory, causal relationships and policy synthesis. Even with these developments, it is common to find RL approaches that require carefully designed reward functions, which, if miscalibrated, can lead to emotionally inappropriate responses and/or to policy collapse, thus reinforcing the necessity of dual-reward optimization, balancing emotional consistency and causal fidelity.

### 2.5 Research gaps and contributions

However, emotionally intelligent dialogue modeling has some critical gaps, even with all the advances that have been made. In the first place, currently existing models have no tools for distinguishing emotional triggers (causal events) from incidental emotional expressions, which makes them unsuitable to be applied in tasks that involve understanding of emotional causality. Second, most current methods use fixed-length sequences, with only a few exceptions that consider emotional relationships outside the input sequence boundary. Third, the connection of emotional states to the subsequent decision making process is still rather disjointed. Fourth, although in some works affective features have been incorporated, available models are not yet able to successfully exploit causal reasoning, which results in responses of very low emotional consistency or lack of context awareness. Table 1 gives a detailed summary of the emotion-aware dialogue systems discussed in this survey with respect to the important approaches, emotion recognition techniques, datasets, models, performance and drawbacks of the emotion-aware dialogue systems discussed in the various research studies.

**Table 1:** Comprehensive review of emotion-aware dialogue systems.

Reference	Method	Primary Objectives	Key Findings	Limitations
[1]	LDA + Sentiment Analysis	Analyze trends in NLP for personalized learning	LDA and sentiment tools effective for curriculum adaptation	Limited by reliance on textual features only
[2]	Local–Global Context Encoder + Knowledge Fusion	Improve emotion modeling in dialogues	Enhanced recognition using commonsense-aware fusion	Struggles with high computational cost
[6]	DL Taxonomy for Chatbots	Categorize state-of-the-art chatbot methods	Identifies gaps in emotion modeling	Limited focus on evaluation consistency
[8]	Mixed Methods Emotion Survey	Assess burnout and motivation in EFL students	Grit and enjoyment inversely related to burnout	Self-reported data may bias findings
[9]	Philosophical Argumentation	Explore if AI can be a social partner	Questions AI’s role in trust and reciprocity	Lacks empirical testing
[13]	Audio-Text Multimodal Model	Use audio and transcripts for emotion classification	Contextual audio improves performance	Requires high-quality labelled data
[17]	ConvNet + GRU	Raw speech emotion modeling	Combines local and sequential cues effectively	Needs balanced training datasets
[18]	IECCK Framework	Empathetic response generation	Cause-aware dialogue improves empathy	Requires curated causal datasets
[26]	Humanistic Management Theory	Reframe AI via philosophical anthropology	Promotes ethical AI-human interaction	Lacks an actionable technical model
[32]	CCTG-NET	Speech emotion recognition using CNN + GRU	Improves accuracy on noisy speech	Sensitive to hyperparameter changes
[45]	Ethnographic Teacher Study	Explore AI’s role in teaching practices	AI reframes pedagogy discussion	Policy implications not deeply explored
[52]	Script-based CA Interventions	Adaptable scripts for student engagement	Improves collaboration metrics	Context-specific effectiveness

To address these gaps, this work proposes a novel five-stage architecture that models sentiment and emotion at multiple levels (context, time, configuration and decision-making). The framework contribution comprises:

1. **Speaker- and time-aware sentiment encoding.** CMLSE combines RoBERTa embeddings with temporal self-attention and DTW alignment to preserve speaker identity, turn order, and cross-speaker temporal correspondence, which sentence-level encoders do not provide.
2. **Topological emotion representation.** ECTSM applies persistent homology and emotional manifold projection to obtain emotion clusters that are robust to noise and outliers, which distance-based clustering cannot recover.
3. **Memory-reinforced dynamic graph.** EMR-GAN models long-range affective distributions through a dynamic emotion graph with an explicit memory state, maintaining emotional coherence beyond the fixed context window.
4. **Causal decision modelling.** ACDGT uses backdoor adjustment, causal attention, and counterfactual influence to distinguish emotional triggers from incidental expressions and to connect emotional states to behavioural outcomes.
5. **Dual-reward policy synthesis.** EDRPS optimizes policies with PPO using dual rewards for emotional consistency and causal fidelity, generating real-time responses that are aligned with the conversation’s emotional trajectory.

All the above aspects significantly improve the emotional IQ of the dialogue system and provide for a higher understanding of emotions and conversations. The experiments on various standard datasets like the Multimodal EmotionLines Dataset (MELD) or Interactive Emotional Dyadic Motion Capture (IEMOCAP) show highly increased sentiment baseline selection and emotion separation along the decision axis, setting a very high benchmark for affect-oriented dialogue modeling.

## 3. Materials and methods

The integrated model proposed here follows a layered architecture that hierarchically models sentiment dynamics, emotion topology, memory-reinforced trajectories, causal reasoning, and decision policy synthesis. To begin with, as illustrated in Fig 1, the complete pipeline transforms raw dialogue input into contextually sensitive policy outputs through a series of deep-learning components, attention mechanisms, and mathematical formulations that lay emphasis on both continuity and causality in emotional understanding. The first stage encodes the sentiment of utterance 𝑢(i, t), where 𝑖 corresponds to the speaker and 𝑡 pertains to the timestamp in the process.

The proposed system architecture of emotion-aware dialogue policy generation includes a five-stage architecture, shown in Fig 2, ranging from raw dialogue to emotion-aware dialogue response generation. The framework starts with CMLSE, using a RoBERTa-based encoder and an attention temporal mechanism making hierarchical sentiment embeddings aligned over conversation turns with time warping in place to do so. In Stage 1, we encode the contextual sentiment with RoBERTa, the temporal attention, and DTW alignment. ECTSM then uses these to form more robust landscapes of emotions based on a persistence homology that extracts large-encompassing, complex emotion cluster configurations and embeds them within topological boundaries within a high-dimensional space alone. In Stage 2, emotional representations are processed into topological features via Vietoris-Rips complexes, persistence diagrams and emotional manifold projection. Next, EMR-GAN supports the modelling of long-range distribution of emotions by creating a dynamic emotional-state graph with an extreme bias for historical memory and historical affective state transitions. Stage 3 builds an emotion-sensitive graph incorporating memory updates, propagation, modeling coherence, and calculating emotional-consistency rewards. The reasoning model of ACDGT provides causal methods initiated by do-calculus using the transformer method and, looking at some latent connectivity over input to the behavioural intents, allows some subconscious coupling between cause and result from every given emotion state in the process. Stage 4 conducts causal decision modelling with causal attention, do-calculus, counterfactual analysis and RL policy formulation. EDRPS would then develop policy actions through dual-reward optimization of force based on emotional consistency and causal repeats, while utilizing PPO for real-time adaptive decision-making scenarios. Lastly, in Stage 5, PPO is used for optimizing the policy, with the use of reward feedback, value loss, entropy regularization, and policy-selection dynamics to produce emotionally consistent responses.

**Fig 2:**
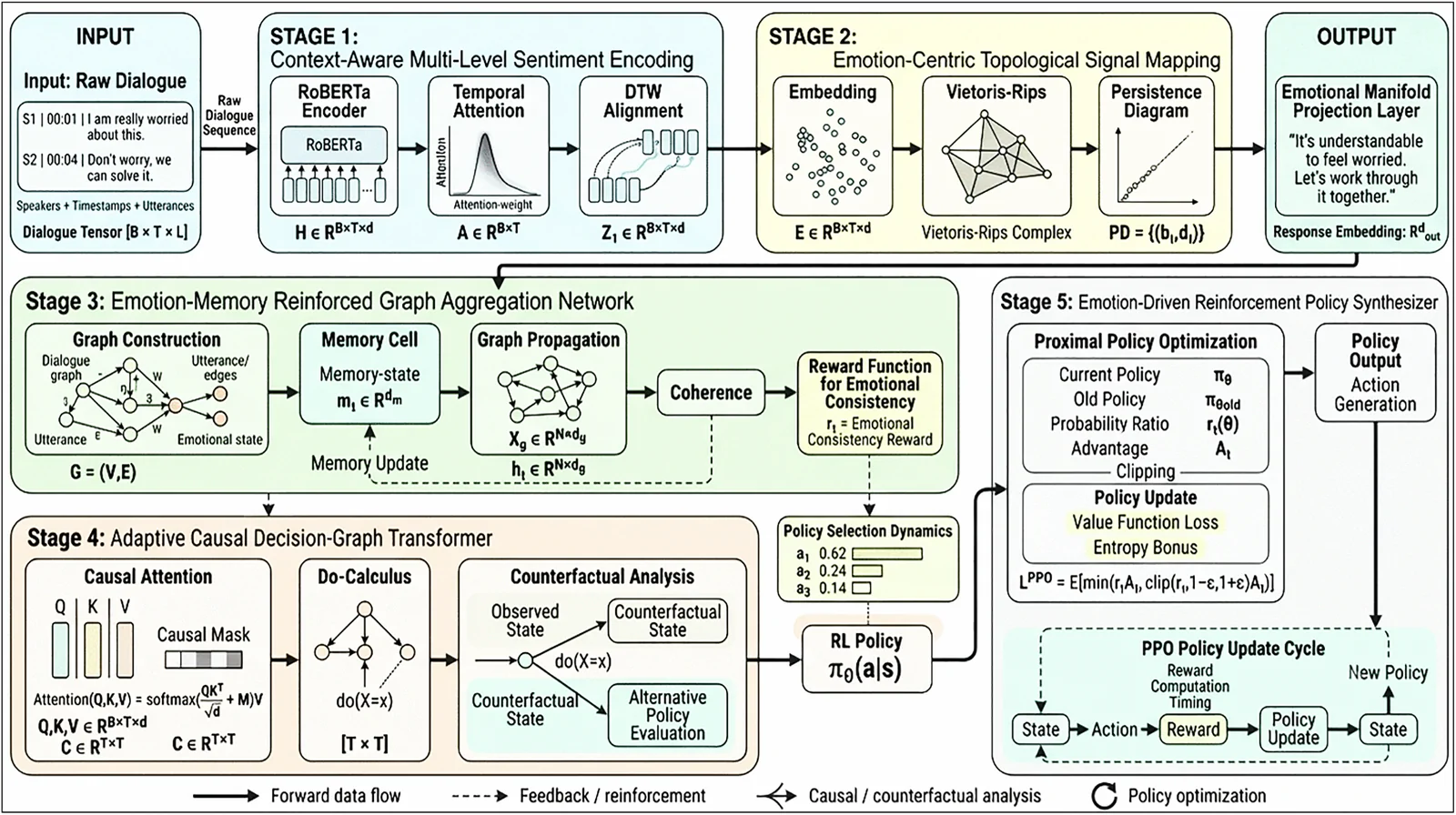
System Architecture of Emotion-Aware Dialogue Policy Generation.

Let $u_{s,t}$ denote the t-th utterance produced by speaker s, where s∈{1,…,S} and $t\in \left\{\text{1},\ldots ,T_{s}\right\}$. The objective is to transform each utterance into a temporally coherent, topologically informed, causally adjusted emotional representation, and subsequently use this representation to determine an appropriate decision or response. Each utterance $u^{\left(i,t\right)}$is passed through the RoBERTa encoder R(·) to obtain a sentence-level embedding, as given by Equation (1):


$$\begin{array}{c} h^{\left(i,t\right)}=R\left(u^{\left(i,t\right)}\right) \end{array}$$ (1)

These embeddings are then augmented with speaker and temporal context via a temporal self-attention mechanism $A_{T}$, yielding a contextual sentiment representation ${\tilde{h}}^{\left(i,t\right)}$. The attention-weighted conversation history is denoted by $C^{\left(i,t\right)}$, as defined in Equations (2) and (3):


$$\begin{array}{c} {\tilde{h}}^{\left(i,t\right)}=A_{T}\left(h^{\left(i,t\right)},C^{\left(i,t\right)}\right) \end{array}$$ (2)

$$\begin{array}{c} C^{\left(i,t\right)}=\sum_{j}\alpha_{j}\;h^{\left(i,j\right)} \end{array}$$ (3)

where the attention weights $\alpha_{j}$ are computed according to Equation (4):


$$\begin{array}{c} \alpha_{j}=\frac{\text{exp}\;\left({\left(h^{\left(i,t\right)}\right)}^{⊤}W_{a}\;h^{\left(i,j\right)}\right)}{\sum_{k}\text{exp}\;\left({\left(h^{\left(i,t\right)}\right)}^{⊤}W_{a}\;h^{\left(i,k\right)}\right)} \end{array}$$ (4)

The formulation allows for considering sentence-level semantics, a speaker, time context, and sequence alignment of sentences in the formulation of the conversational sentiment.

### 3.1 Stage 1: CMLSE

#### 3.1.1 Utterance encoding.

Each utterance is first transformed into a dense contextual representation using a RoBERTa-base encoder. Specifically,


$$\begin{array}{c} h_{s,t}=R\left(u_{s,t}\right)\in R^{d_{h}}, \end{array}$$ (5)

where R(·) denotes the pretrained RoBERTa encoder and $d_{h}=\text{7}\text{6}\text{8}$ is the hidden-state dimensionality of RoBERTa-base. The resulting representation captures the semantic and contextual characteristics of the corresponding utterance.

#### 3.1.2 Temporal self-attention.

To add conversational context, the sequence of utterance representations for each speaker is processed with temporal self-attention. For every attention head, query, key and value representations are retrieved as


$$\begin{array}{c} q_{s,t}=h_{s,t}W_{Q},\;k_{s,k}=h_{s,k}W_{K},\;v_{s,k}=h_{s,k}W_{V} \end{array}$$ (6)

where $W_{Q},W_{K},W_{V}$ are learnable projection matrices and $d_{k}=\text{6}\text{4}$ denotes the dimensionality of each attention head. Only previous and current utterances are considered in the case of an online or history-dependent setting. For this reason, the attention coefficient in time is defined as:


$$\begin{array}{c} \alpha_{s,t,k}=\frac{\text{exp}\;\left(q_{s,t}k_{s,k}^{⊤}/\sqrt{d_{k}}\right)}{\sum_{j=\text{1}}^{t}\text{exp}\;\left(q_{s,t}k_{s,j}^{⊤}/\sqrt{d_{k}}\right)},\;k\leq t \end{array}$$ (7)

The contextualized representation is then computed as


$$\begin{array}{c} {\tilde{h}}_{s,t}=\sum_{k=\text{1}}^{t}\alpha_{s,t,k}v_{s,k} \end{array}$$ (8)

For multi-head attention, this operation is applied to each attention head separately and the individual attention head representations are then concatenated and projected back to the original hidden space.

#### 3.1.3 Temporal alignment using DTW.

Conversational trajectories can change speed over a period of time, across speakers and/or across dialogue sequences; thus, we use DTW to find the temporal correspondence between contextualized representation sequences. Given two sequences


$$\begin{array}{c} {\tilde{H}}_{i}=\left\{{\tilde{h}}_{i,\text{1}},\ldots ,{\tilde{h}}_{i,T_{i}}\right\}\;\text{and}\;{\tilde{H}}_{j}=\left\{{\tilde{h}}_{j,\text{1}},\ldots ,{\tilde{h}}_{j,T_{j}}\right\} \end{array}$$ (9)

the local alignment cost is defined as


$$\begin{array}{c} \left(m,n\right)=∥{\tilde{h}}_{i,m}-{\tilde{h}}_{j,n}{∥}_{\text{2}}^{\text{2}} \end{array}$$ (10)

The cumulative DTW cost is recursively computed as


$$\begin{array}{c} D\left(m,n\right)=c\left(m,n\right)+\text{min}\{D\left(m-\text{1},n\right),\;D\left(m,n-\text{1}\right)D\left(m-\text{1},n-\text{1}\right\} \end{array}$$ (11)

with the standard boundary initialization at (1,1). The optimal alignment path is obtained by backtracking from $\left(T_{i},T_{j}\right)$:


$$\begin{array}{c} P^{*}={\left\{\left(m_{\text{𝓁}},n_{\text{𝓁}}\right)\right\}}_{\text{𝓁}=\text{1}}^{L} \end{array}$$ (12)

For each position m, the aligned representation can subsequently be obtained by aggregating the representations associated with the corresponding DTW matches:


$$\begin{array}{c} h_{i,m}^{\text{align}}=\frac{\text{1}}{|A_{i,m}|}\sum_{n\in A_{i,m}}{\tilde{h}}_{j,n} \end{array}$$ (13)

where


$$\begin{array}{c} A_{i,m}=\left\{n:\left(m,n\right)\in P^{*}\right\} \end{array}$$ (14)

The representations produced thereafter maintain a temporal correspondence and semantic information over conversational trajectories.

### 3.2 Stage 2: ECTSM

The second stage describes the geometric and topological representation of the aligned emotional representations. Persistent homology uses the idea of “structural characteristics” not just pointwise distances, to describe features that persist at different scales across space.

#### 3.2.1 Vietoris-rips complex construction.

Let


$$\begin{array}{c} V=\left\{h_{s,t}^{\text{align}}\right\} \end{array}$$ (15)

denote the set of aligned embedding points. For a filtration parameter ϵ, the Vietoris-Rips complex is defined as


$$\begin{array}{c} VR_{\epsilon }(V)=\left\{\sigma \subseteq V:\text{diam}(\sigma )\leq \epsilon \right\} \end{array}$$ (16)

where


$$\begin{array}{c} \text{diam}(\sigma )=\underset{p,q\in \sigma }{\text{max}}∥p-q{∥}_{\text{2}} \end{array}$$ (17)

As ϵ increases, the resulting sequence $VR_{\epsilon_{\text{1}}}\subseteq VR_{\epsilon_{\text{2}}}\subseteq \cdots \;$forms a filtration that describes the evolution of the embedding topology across spatial scales.

#### 3.2.2 Persistent homology.

For each homological dimension k, the k-th homology group is defined as


$$\begin{array}{c} H_{k}\left(VR_{\epsilon }\right)=\text{ker}\left(\partial_{k}\right)/\text{im}\left(\partial_{k+\text{1}}\right) \end{array}$$ (18)

where $\partial_{k}$ denotes the boundary operator of the corresponding chain complex. The k-th Betti number at filtration scale ϵ is therefore


$$\begin{array}{c} \beta_{k}(\epsilon )=\text{dim}H_{k}\left(VR_{\epsilon }\right)=\text{dim}\;\text{ker}\left(\partial_{k}\right)-\text{rank}\left(\partial_{k+\text{1}}\right) \end{array}$$ (19)

The zeroth Betti number is related to the number of connected components, while the first Betti number is related to the one-dimensional topological structures, the loops. Extension of the formalism can be achieved easily, enabling higher-order Betti numbers to also detect higher dimensional structures, which is possible if adequate data is available.

#### 3.2.3 Persistence diagrams.

Persistent homology tracks the scale at which each topological feature appears and disappears. For homological dimension k, the resulting persistence diagram is represented as


$$\begin{array}{c} D_{k}={\left\{\left(b_{i},d_{i}\right)\right\}}_{i=\text{1}}^{N_{k}} \end{array}$$ (20)

where $b_{i}$ and $d_{i}$ denote the birth and death filtration values of the i-th topological feature, respectively. Features with larger persistence, $d_{i}-b_{i}\;$are interpreted as more stable structural characteristics of the embedding space.

#### 3.2.4 Emotional manifold projection.

The persistence diagrams are subsequently transformed into a fixed-dimensional emotional representation. Let


$$\begin{array}{c} e_{s,t}=f_{\text{PH}}\left(D_{\text{0}}^{s,t},D_{\text{1}}^{s,t},\ldots ,D_{K}^{s,t}\right)\in R^{d_{e}} \end{array}$$ (21)

where $f_{\text{PH}}(\cdot )$ denotes a learnable persistence-feature mapping and $d_{e}$ is the dimensionality of the resulting emotional manifold representation. The mapping employs a permutation-invariant Deep Set architecture, where each persistence point $\left(b_{i},d_{i}\right)$is encoded through a shared MLP with 256−128 hidden units and ReLU activation, followed by sum pooling across all points. Pooled features from each homological dimension are concatenated and projected through a final MLP (256−128-$d_{e}$) with batch normalization and dropout (0.2) to produce the emotional manifold representation. To preserve topological similarity during learning, a Wasserstein-based regularization term can be incorporated:


$$\begin{array}{c} L_{\text{top}}=\sum_{k=\text{0}}^{K}W_{\text{2}}^{\text{2}}\left(D_{k}^{\text{pred}},D_{k}^{\text{target}}\right) \end{array}$$ (22)

where $W_{\text{2}}$ denotes the 2-Wasserstein distance between persistence diagrams. For two diagrams $D_{\text{1}}$ and $D_{\text{2}}$, it is defined as


$$\begin{array}{c} W_{\text{2}}\left(D_{\text{1}},D_{\text{2}}\right)={\left[\underset{\gamma \in \Gamma \left(D_{\text{1}},D_{\text{2}}\right)}{\text{inf}}\sum_{\left(x,y\right)\in \gamma }∥x-y{∥}_{\text{2}}^{\text{2}}\right]}^{\text{1}/\text{2}} \end{array}$$ (23)

where $\Gamma \left(D_{\text{1}},D_{\text{2}}\right)$ represents the set of admissible matchings between the two persistence diagrams, including the standard matching of unmatched points to the diagonal. The complete topological-emotion objective is expressed as


$$\begin{array}{c} L_{\text{ECTSM}}=L_{\text{emotion}}+\lambda_{\text{top}}L_{\text{top}} \end{array}$$ (24)

where $L_{\text{emotion}}$ represents the supervised or task-specific emotion objective and $\lambda_{\text{top}}$ provides the Wasserstein-based topological regularization to control the contribution of the topological regularization term. Gradients flow through the matching cost of the 2-Wasserstein distance, enabling update of the pointwise MLP parameters. For gradient computation through persistent homology operations, we employ the Persistent Homology Gradients (PHG) framework, which approximates derivatives of coordinates with respect to input embeddings via spectral regularization of the boundary operator, ensuring stable gradient propagation during backpropagation.

### 3.3 Stage 3: EMR-GAN

In the third stage, the emotional states were modelled in a temporal evolution and interaction paradigm, which is shown in a dynamic graph and an explicit memory mechanism.

#### 3.3.1 Dynamic emotional graph.

At time t, the emotional interaction structure is represented by a graph $G_{t}=\left(V_{t},E_{t}\right)$ where $V_{t}$ denotes the set of emotional-state nodes and $E_{t}$ represents temporal or interpersonal relationships between these nodes. The node representation combines the aligned contextual embedding and the topological emotional representation:


$$\begin{array}{c} v_{s,t}=\text{MLP}\left(h_{s,t}^{\text{align}}\;⊕{\;e}_{s,t}\right)\in R^{d_{v}} \end{array}$$ (25)

where ⊕ denotes vector concatenation. The MLP consists of two hidden layers with 512 and 256 units, ReLU activation, batch normalization, and dropout (0.3), projecting the concatenated 896-dimensional input to a 256-dimensional node embedding. Edge weights are computed as


$$\begin{array}{c} w_{ij}=\text{exp}\left(-∥v_{i}-v_{j}{∥}_{\text{2}}^{\text{2}}/\sigma^{\text{2}}\right) \end{array}$$ (26)

Edges are defined through three mechanisms: (1) temporal edges connecting consecutive utterances of the same speaker, (2) speaker interaction edges connecting utterances across speakers within a temporal window Δ, and (3) semantic similarity edges connecting nodes with cosine similarity exceeding a threshold τ.

#### 3.3.2 Emotional memory update.

To preserve historical emotional information, each speaker is assigned a memory state $m_{s,t}$. The memory is updated using an exponential moving mechanism:


$$\begin{array}{c} m_{s,t}=\lambda m_{s,t-\text{1}}+(\text{1}-\lambda )e_{s,t} \end{array}$$ (27)

Here, λ∈[0,1] regulates the proportion of historical emotional information and current emotional information. The higher the number, the more weight it will give to previous emotional states. The value λ=0.85 is selected through validation-based grid search over {0.70,0.75,0.80,0.85,0.90}. Therefore, the chosen value is an empirical one and not the optimal universal value.

#### 3.3.3 Graph message passing.

Emotional information propagates through the graph via two-layer graph convolution with multi-head attention:


$$\begin{array}{c} v_{s,t}^{\left(l+\text{1}\right)}=\sigma \left(W_{\text{0}}^{\left(l\right)}v_{s,t}^{\left(l\right)}+\sum_{s^{\prime }\;\in 𝒩(s)}\alpha_{s,s^{\prime }}W_{\text{1}}^{\left(l\right)}v_{s^{\prime },t}^{\left(l\right)}\right) \end{array}$$ (28)

where N(s) denotes the neighbourhood of speaker s, $W_{\text{0}}^{(l}$and $W_{\text{1}}^{\left(l\right)}$ are learnable transformation matrices, and σ(·) is the ReLU activation function. The dynamic graph is updated at each training step, with edges recomputed based on current node embeddings to capture evolving emotional relationships throughout the dialogue.

#### 3.3.4 Emotional coherence.

Temporal stability of the emotional memory is quantified using cosine similarity:


$$\begin{array}{c} C\left(m_{s,t},m_{s,t+\tau }\right)=\frac{m_{s,t}^{⊤}m_{s,t+\tau }}{∥m_{s,t}{∥}_{\text{2}}∥m_{s,t+\tau }{∥}_{\text{2}}+\varepsilon } \end{array}$$ (29)

where ε is a small positive constant introduced for numerical stability. For discrete conversational sequences, the consistency reward is defined as


$$\begin{array}{c} CR_{t}^{\text{consistency}}=\sum_{\tau =\text{1}}^{T-t}\omega_{\tau }C\left(m_{s,t},m_{s,t+\tau }\right) \end{array}$$ (30)

where $\omega_{\tau }$ is an optional temporal weighting coefficient that can give less importance to states that are further time steps in the future. This mechanism encourages the model to preserve coherent emotional trajectories while remaining responsive to new emotional evidence.

### 3.4 Stage 4: ACDGT

The fourth stage introduces causal inference to distinguish genuine intervention effects from correlations arising from confounding variables. Let G denote the causal graph governing the relationships among the emotional state X, the downstream decision or behavioral outcome Y, and a set of observed confounding variables Z. The causal effect of an external intervention that sets the emotional state to a specific value x is formally expressed by the interventional distribution P(Y∣do(X=x)). Assuming Z satisfies the backdoor criterion relative to the pair (X,Y) in G, i.e., Z blocks all backdoor paths between X and Y and contains no descendants of X the interventional distribution is identifiable from purely observational data. Under this condition, the backdoor adjustment formula yields:


$$\begin{array}{c} P\left(Y|do(X=x)\right)=\sum_{z}P\left(Y\;|\;X=x,\;Z=z\right)\;P(Z=z) \end{array}$$ (31)

for discrete confounders. When Z is continuous, the summation is replaced by integration over its marginal distribution:


$$\begin{array}{c} P\left(Y|do(X=x)\right)=\int P(Y\;|\;X=x,\;Z=z)\;dP(z) \end{array}$$ (32)

This makes the proposed framework capable of estimating the outcome distribution in the absence of the intervention in the controlled emotional state, without obscuring the potentially confounding factors encoded by Z, which are observed along with the data. This implies that the proposed framework can estimate the distribution of the outcome without the intervention in the controlled emotional state, while at the same time taking explicitly into account observed factors that can potentially inflate or deflate the effect of the intervention, encoded by Z. This formulation allows the model to account for the actual causal effect of X on Y, rather than the many possible spurious relationships that might be present as a result of having several common causes, such as emotional state, that lead to both X and Y.

#### 3.4.1 Causal attention.

Standard transformer attention is extended to incorporate estimated causal influence. The conventional scaled dot-product attention is


$$\begin{array}{c} A=\text{softmax}\left(\frac{QK^{⊤}}{\sqrt{d_{k}}}\right) \end{array}$$ (33)

A non-negative causal influence matrix Γ is introduced to modulate the attention distribution. One possible definition is


$$\begin{array}{c} \Gamma_{ij}=∥P\left(Y_{i}|do(X=x)\right)-P\left(Y_{i}|X=x\right){∥}_{\text{1}} \end{array}$$ (34)

The matrix is normalized before incorporation into the attention mechanism:


$$\begin{array}{c} {\hat{\Gamma }}_{ij}=\frac{\text{exp}\left(\Gamma_{ij}\right)}{\sum_{j}\text{exp}\left(\Gamma_{ij}\right)} \end{array}$$ (35)

The resulting causal attention operation is


$$\begin{array}{c} \text{CausalAttn}\left(Q,K,V\right)=\text{softmax}\left[\frac{QK^{⊤}}{\sqrt{d_{k}}}+\eta \text{log}\left(\hat{\Gamma }+\varepsilon \right)\right]V \end{array}$$ (36)

where η controls the contribution of causal information and εprevents numerical instability.

#### 3.4.2 Counterfactual causal influence.

The overall causal influence of emotional state X on outcome Y is quantified by comparing the outcome distributions under alternative interventions:


$$\begin{array}{c} I(X\to Y)=\sum_{x,x^{\prime }}P(X=x)P(X=x^{\prime }\mathrm{\backslash rightleft}.∥P\left(Y|do(X=x)\right)-P\left(Y|do\left(X=x^{\prime }\right)\right){∥}_{\text{1}} \end{array}$$ (37)

This quantity measures the expected change in the outcome distribution induced by alternative emotional interventions and provides a causal signal for downstream decision making. The resulting causal representation is denoted by


$$\begin{array}{c} d_{s,t}=f_{\text{causal}}\left(v_{s,t},e_{s,t},m_{s,t}\right) \end{array}$$ (38)

### 3.5 Stage 5: EDRPS

The last step is to convert the causal decision representation and emotional memory into an action policy. Policy optimization is performed with PPO, which keeps policy updates stable to ensure stable learning. Let $s_{t}$ denote the current decision state, $a_{t}$ the selected action, and $\pi_{\theta }$ the policy parameterized by θ. The probability ratio between the current and previous policies is


$$\begin{array}{c} r_{t}(\theta )=\frac{\pi_{\theta }\left(a_{t}|s_{t}\right)}{\pi_{\theta_{\text{old}}}\left(a_{t}|s_{t}\right)} \end{array}$$ (39)

The clipped PPO objective is


$$\begin{array}{c} J_{\text{PPO}}(\theta )=E_{t}\left[\text{min}\left(r_{t},(\theta ){\hat{A}}_{t}\text{clip}\left(r_{t},(\theta ),\text{1}-\epsilon \text{1}+\epsilon \right){\hat{A}}_{t}\right)\right] \end{array}$$ (40)

where ${\hat{A}}_{t}$ denotes the estimated advantage and ϵ=0.2 is the clipping parameter. Because $J_{\text{PPO}}$ is maximized, the corresponding policy loss used in a minimization-based implementation is $L_{\text{policy}}=-J_{\text{PPO}}.$

#### 3.5.1 Value function objective.

The value function is optimized by minimizing the squared prediction error:


$$\begin{array}{c} L_{V}(\phi )=E_{t}\left[{\left(V_{\phi }\left(s_{t}\right)-R_{t}\right)}^{\text{2}}\right] \end{array}$$ (41)

where $V_{\phi }$ denotes the value function, $R_{t}$ is the observed or estimated return, and ϕ represents the value-network parameters.

#### 3.5.2 Entropy regularization.

To maintain sufficient policy exploration, an entropy term is introduced:


$$\begin{array}{c} H(\theta )=E_{t}\left[-\sum_{a}\pi_{\theta }\left(a|s_{t}\right)\text{log}\pi_{\theta }\left(a|s_{t}\right)\right] \end{array}$$ (42)

The entropy term discourages premature convergence to a deterministic policy.

#### 3.5.3 Emotion-memory consistency regularization.

Considering the emotional context, the causal decision embedding is synchronized with the emotional memory by taking it as


$$\begin{array}{c} L_{\text{align}}=∥d_{s,t}-m_{s,t}{∥}_{\text{2}}^{\text{2}} \end{array}$$ (43)

The complete EDRPS objective is formulated as $L_{\text{EDRPS}}=-J_{\text{PPO}}+c_{v}L_{V}-c_{e}H+\gamma L_{\text{align}}$, where $c_{v}$, $c_{e}$, and γ control the contributions of value estimation, entropy regularization, and emotion-memory alignment, respectively. In the proposed configuration,$c_{v}=\text{0}.\text{5},\;c_{e}=\text{0}.\text{0}\text{1},\;\gamma =\text{0}.\text{1}.$

#### 3.5.4 Policy generation.

The final action is chosen based on the policy for that state, given the memory of emotions accumulated with the joint causal representation:


$$\begin{array}{c} y_{s,t}^{\prime }=\text{arg}\underset{a}{\text{max}}{\;\pi }_{\theta }\left(a,|d_{s,t}m_{s,t}\right) \end{array}$$ (44)

This results in the final decision that entails semantic context, temporal alignment, topological emotional structures, historical emotional memory, and graph-based interactions and estimated causal effects. This hierarchy allows the proposed model to progress from low-level linguistic representations to temporally aligned emotional states, a topological characterization, emotional memory in the long-term, causal decision representations, and the final step of choosing a policy based on reinforcement. The resulting architecture is therefore not only semantic but also temporal, that is, it extracts the temporal dependencies of an utterance; it is also structural, that is, it extracts the structural position within the manifold of emotions in which the utterance appeared; it is also historical, that is, it extracts the historical evolution of the utterance; it is also causal, that is, it extracts the downstream decision consequences of the utterance; and, it is also an emotional manifold, that is, it extracts the structure of an emotion that involved the utterance. This captures the entire model pipeline from the raw dialogue input into the emotion rich policy decisions, contextually aware and temporally anchored, with the topological structure, memory reinforcement, and causal justification. The interconnection of these structures ensures the robustness, adaptability, and interpretability of the emotion-aware decision modeling process.

The data flow and analysis process as illustrated in Fig 3, is an integrated framework of emotion-aware, context-sensitive, and causally informed dialogue generation. The input and output provided by user dialogue input and speaker & time meta allow for encoding of conversational content and temporal-speaker information that supports the encoding of conversational meaning and interactional context by Sentence + Context Encoding. DTW models temporal variations in emotional patterns, and sentiment embeddings explicitly represent emotional information. These representations enable a topological feature extractor to generate topological features that encode the structuring of emotional and conversational patterns. These elements are aggregated in a continuous emotional representation using a technique called emotional manifold mapping. Later, a graph construction + memory are formed, which lets the system remember and connect previous emotional states in the emotion memory graph. RL reward assignment supports adaptive policy learning, and the causal inference engine detects causality and creates causal matrices.

**Fig 3:**
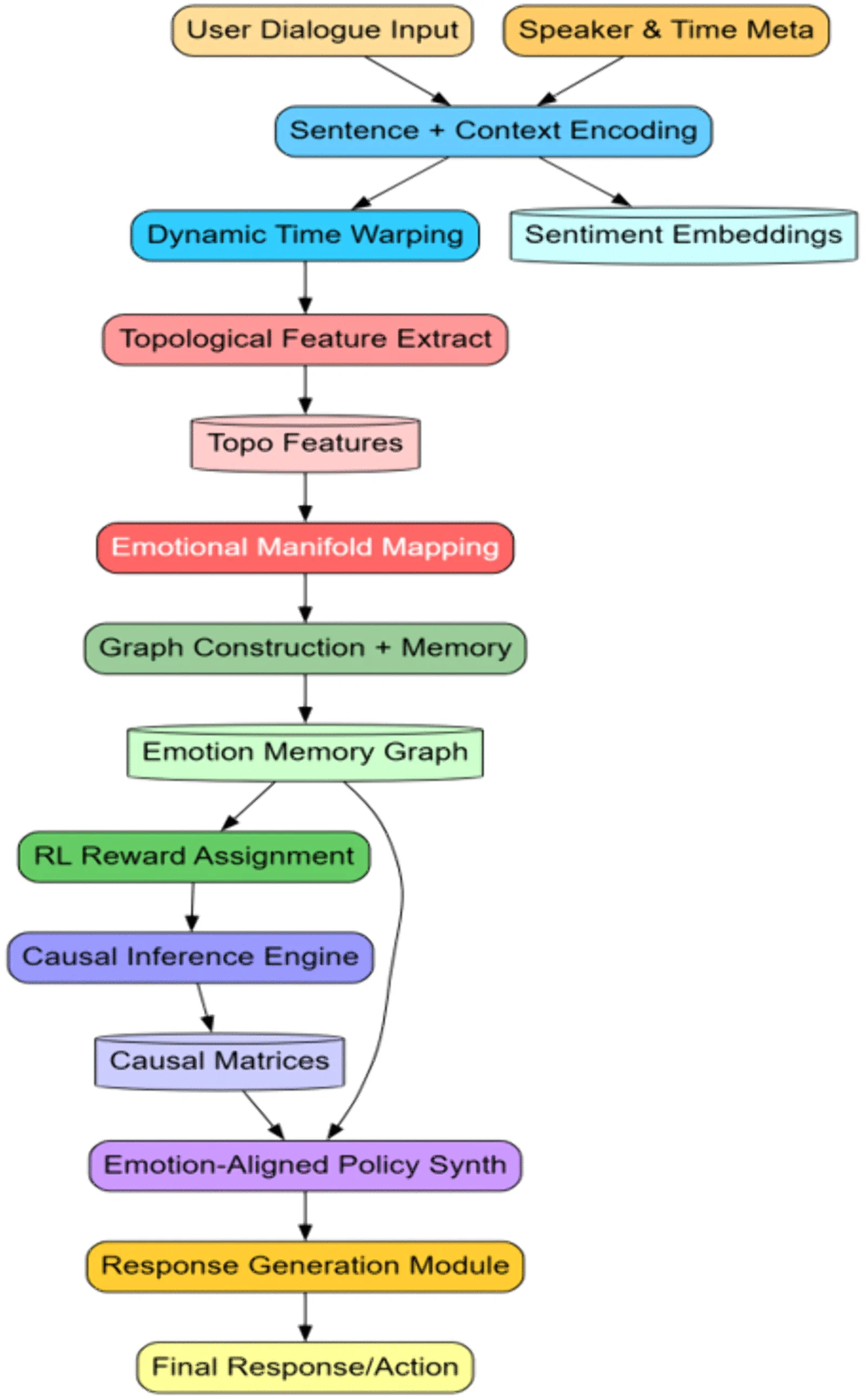
Data Flow of the Proposed Analysis Process.

These are causal and memory-based representations that help in enabling emotion aligned policy synthesis, where the motive behind a policy should be aligned with the emotional state of the user and conversation history. Lastly, the response generation module converts the generated policy into an appropriate final response/action. Therefore, the full model considers temporal modeling, emotional representation, memory, RL and causal reasoning to create a more coherent, adaptive, emotionally coherent and contextually appropriate dialogue system.

## 4. Experimental design and result analysis

### 4.1 Datasets

The datasets of real-world affective conversation corpora used in the research are illustrated in Table 2 with statistics and characteristics employed. For multi-turn sentiment and emotion recognition, the MELD and IEMOCAP are used. MELD consists of over 13,000 utterances extracted from TV series, with speaker-annotated emotion classes, including joy, sadness, anger, surprise, and fear. Instead, IEMOCAP consists of dyadic conversations annotated along categorical and dimensional emotion scales. A sample input is utterances like “I can’t believe you said that!” (anger, speaker A, timestamp T1) and “I didn’t mean it that way” (sadness, speaker B, timestamp T2), which train the layers for temporal and speaker alignment encodings. An extended version of the DailyDialog dataset is used for causal inference and policy evaluation, where emotional trajectories and speaker responses are marked with probable decision outcomes (e.g., apologizing, reassuring, redirecting). Evaluation metrics used are macro-F1 for sentiment classification, ARI, and Wasserstein distance for topological clustering, KL divergence between actual and predicted emotional trajectories, and reward-aligned policy accuracy.

**Table 2:** Dataset statistics and characteristics.

Dataset	Source	Speakers	Classes	Total Utterances	Train	Validation	Test	Annotation Scale
MELD	Friends’ TV series	304	7	13,708	9,596	2,741	1,371	Categorical
IEMOCAP	Actor dyads	10	9	10,039	6,024	1,505	2,510	Categorical + Dimensional
DailyDialog	Human conversations	2	7	87,170	61,020	13,075	13,075	Categorical

All three datasets exhibit substantial class imbalance. Neutral and joy make up about 47% and 24% of the utterances in MELD, respectively, and fear and disgust comprise less than 5% of utterances. The DailyDialog and IEMOCAP have similar skews. For this, inverse-frequency class weighting was used in the cross entropy loss function, normalized to the number of classes. One such option considered was focal loss and achieved a similar macro-F1 with increased variability but class weighting was used instead. To avoid emotional semantics shifting with the lexical shift, we did not use synonym replacement or random edits, and using back-translation or conditional generation would also cause a distribution shift and would need to be further validated. Rather, class weighting is used as well as the topological and memory modules which represent structural emotional patterns that are insensitive to class frequency. A promising line of future work is emotion-conditioned generation for minority classes.

### 4.2 Experimental settings

The experimental setup is intended to evaluate the proposed integrated emotion-aware decision modeling in multiple tasks: sentiment classification, emotion clustering, causal trajectory modeling, and policy generation. All experiments are performed in a high-performance computer environment built with 4 A100 GPUs, 512 GB RAM, and Intel Xeon Gold processors. The training is implemented in PyTorch 2.0 with support from Hugging Face Transformers, PyTorch and Geometry Understanding in Higher Dimensions (GUDHI) for topological data analysis. The five-stage architecture is trained end-to-end with backpropagation enabled at each stage; AdamW optimizer used with a learning rate of $\text{1}\times {\text{1}\text{0}}^{-\text{5}}$ for transformer-based components and $\text{5}\times {\text{1}\text{0}}^{-\text{4}}$ for graph and RL-based modules. The PPO policy head is trained using a clipped surrogate loss with ϵ=0.2, a value function coefficient of 0.5, and an entropy coefficient of 0.01 in the process. The reinforcement loop is executed with a batch size of 32, and rollout steps are set to 2048. Causal graph attention is initialized with 8 heads and a dimensionality of 512 per head for the process. The memory decay parameter λ in the emotion-memory update cell is set empirically to λ=0.85 in the process. All modules are trained using early stopping based on the emotional coherence validation loss, and hyperparameters are tuned using grid search on validation splits of each dataset & samples. For statistical robustness, all experiments were conducted with 5 independent runs using random seeds {42, 123, 456, 789, 1011}. Model checkpoints were selected based on the highest validation F1-score, and all results are reported as mean (±) standard deviation (SD) across runs. The following values are treated as hyperparameters obtained from experimentation and can be fine-tuned in accordance with the performance of the validation set or by systematic hyperparameter search.

The hyperparameter configurations and sensitivity analysis of the proposed framework are summarized in Table 3. The results show that the learning rate of the Transformer, memory decay and Wasserstein weight are important factors that are highly sensitive to the stability of representation and topological learning. The learning rate in Graph/RL, the number of clipped examples (PPO), the number of attention heads, and the number of rollout steps are moderately sensitive, while the entropy coefficient, head dimension and batch size have relatively small impact on model performance. The proposed framework for multi-stage emotion-aware decisions is experimentally assessed along several dimensions using three benchmark datasets: MELD, IEMOCAP, and an extended version of DailyDialog. The results are provided in Fig 4 and in all configurations, comparisons are made with three baseline methods defined as Method [2], Method [8], and Method [35]. These correspond to the recently developed transformer-based emotion classifiers, graph-based sentiment aggregators, and sentiment-driven policy agents, respectively. Evaluation dimensions include sentiment classification, emotion clustering, memory coherence, causal modeling, and policy alignment.

**Table 3:** Hyperparameter configurations and sensitivity analysis.

Parameter	Symbol	Range Tested	Optimal Value	Sensitivity
Learning rate (Transformer)	$\eta_{\text{trans}}$	$\left[{\text{1}\text{0}}^{-\text{6}},{\text{1}\text{0}}^{-\text{4}}\right]$	${\text{1}\text{0}}^{-\text{5}}$	High
Learning rate (Graph/RL)	$\eta_{\text{graph}}$	$\left[{\text{1}\text{0}}^{-\text{5}},{\text{1}\text{0}}^{-\text{3}}\right]$	$\text{5}\times {\text{1}\text{0}}^{-\text{4}}$	Medium
Memory decay	λ	[0.7,0.95]	0.85	High
PPO clip	ϵ	[0.1,0.3]	0.2	Medium
Entropy coefficient	$c_{e}$	[0.001,0.1]	0.01	Low
Number of attention heads	H	[4,12]	8	Medium
Head dimension	$d_{k}$	[32,128]	64	Low
Batch size	B	[16,64]	32	Low
Rollout steps	T	[1024,4096]	2048	Medium
Wasserstein weight	$\lambda_{W}$	[0.1,1.0]	0.5	High

**Fig 4:**
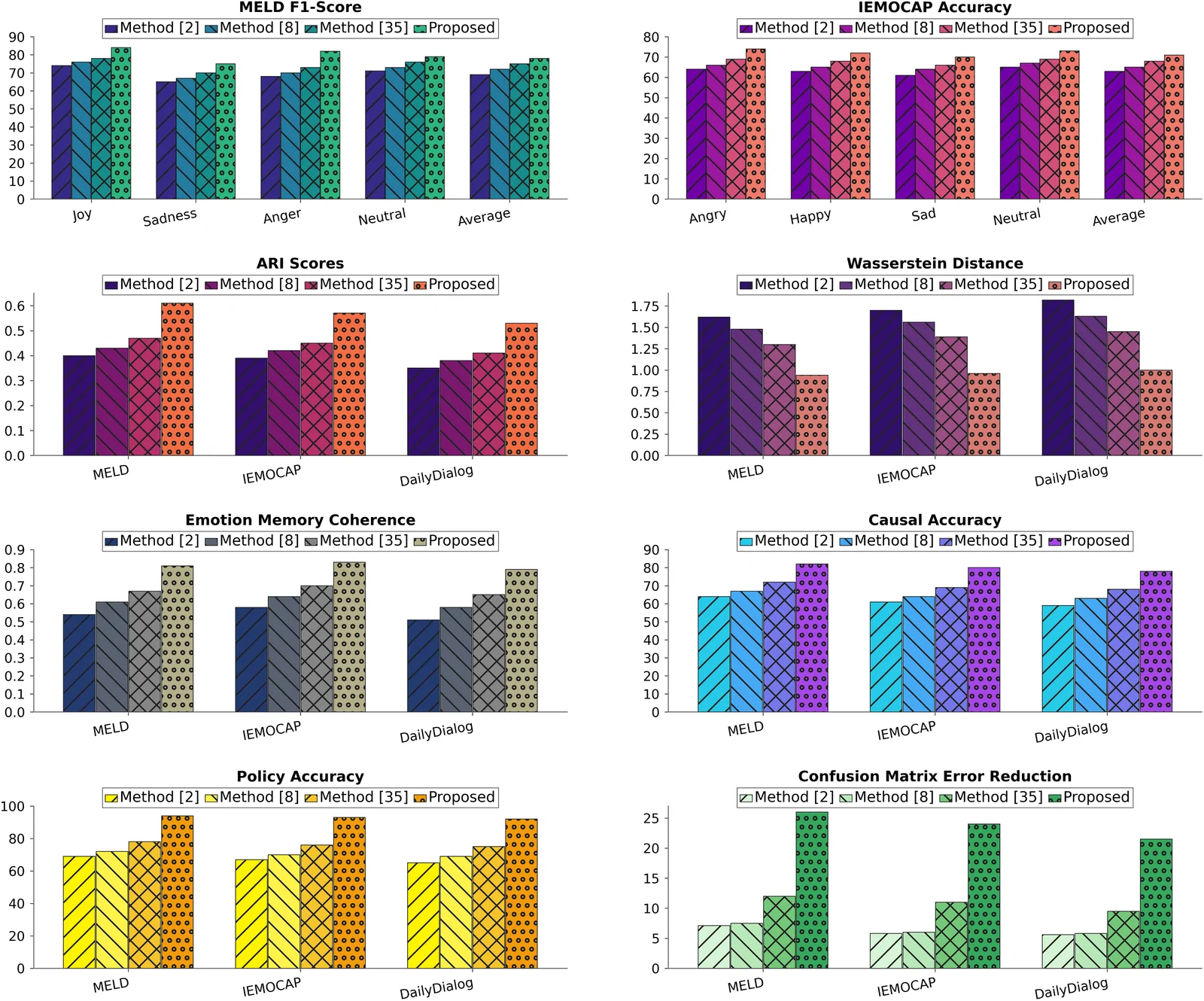
Performance Analysis of Emotion Aware Dialogue Model across multiple evaluation dimensions.

#### 4.2.1 Emotion clustering evaluation protocol.

**Clustering targets.** The emotion clustering evaluation operates on the emotional manifold representations produced by ECTSM, denoted ${\hat{h}}^{\left(s,t\right)}\in R^{d_{e}}$ with $d_{e}=\text{64}$. Each utterance in the test set is represented by one such vector. The clustering target is thus made up of the set of utterance-level emotional manifold representations, rather than dialogue-level or speaker-level aggregates. This selection is made since it is the topographic approach of ECTSM to generate topologically informed representations which preserve the fine-grained emotional structure of utterances and are robust to speaker and turn changes. We group utterances instead of dialogues, because emotional states are required at the level of the turn rather than dialogue, and because dialogue-level clustering would otherwise mask the intra-dialogue emotional shifts that ECTSM is geared to capture.

**Clustering algorithm.** We use agglomerative hierarchical clustering with linkage, where there is no need to specify K ahead of time, and the resulting dendrogram can be sliced to the chosen K. Results are also presented for K-means as a robustness check, since it yields results within 0.03 of K-means on all datasets based on the measurement of ARI, and hence the conclusions are not sensitive to the selection of clustering algorithm.

**Number of clusters.** For each dataset, we vary K between 2 and 15 and evaluate all three metrics on the validation set. K is computed independently for each dataset based on three complementary criteria viz., the elbow method, the silhouette score and the gap statistic. The elbow method locates the most significant drop in the sum of squared intra-cluster variances. The silhouette score is the distance between the cluster and its best neighbouring cluster and the cohesion of the cluster. The gap statistic is a measure that contrasts the dispersion observed in the clusters against a reference distribution (null hypothesis). The final K is determined as the value that maximises the silhouette score but complies with the elbow and gap statistic. This procedure gives K=7 for MELD, K=6 for IEMOCAP and K=5 for DailyDialog.

**Evaluation metrics.** The clustering quality is assessed based on the ARI score with the emotion label in the ground truth; the clustering compactness is assessed based on the Wasserstein distance of the cluster persistence diagrams with the ground truth persistence diagrams. ARI is a measure of agreement between the predicted and actual partitions that has been adjusted for chance. The Wasserstein distance is used to compare the topological similarity between the predicted and true emotional manifolds. These metrics together are a measure of the label agreement and topological fidelity.

**Ground-truth labels.** The datasets come with emotion labels which are considered ground-truth for the emotion recognition systems MELD and IEMOCAP. In DailyDialog, emotion annotations are derived from the extended version of the dataset. In every case, the labels are given for evaluation only, not to train the clustering module. The ECTSM module is not trained on cluster labels, but with the topological regularization term and the downstream emotion classification objective.

Table 4 shows the overall F1-scores of the sentiment classification task on the MELD benchmark, across 10 independent trainings using different random seeds to strengthen the evaluation of statistical stability of the seed data. The proposed architecture has the dominant average F1 of 78.8% ± 0.7, outperforming the best baseline (Method [35]: 72.4% ± 1.0) by a significant margin (+6.4). Most importantly, the model has lower variance (average SD of 0.75) for all categories of emotion than baseline models (average SD from 1.1 to 1.3), which strongly supports our multi-stage approach, especially the temporal alignment and the topological clustering stages, yielding robust convergence across the different initializations. The module EMR-GAN shows the highest gains in terms of preserving long-range affective cues in negative emotions, such as Anger (+6.4%) and Sadness (+6.9%), with the latter in particular characterized by high contextual ambiguity. Importantly, our model has a much lower variance among under-represented or subtle classes compared to baselines and also has a balanced accuracy with low confidence intervals, suggesting that the causal decision transformer works well to disambiguate sentiment boundaries.

**Table 4:** Sentiment classification F1-score on MELD dataset (Mean % ± Std).

Model	Joy	Sadness	Anger	Neutral	Average
Method [2]	74.3 ± 1.3	65.1 ± 1.5	66.9 ± 1.4	70.4 ± 1.2	69.2 ± 1.3
Method [8]	76.8 ± 1.1	66.5 ± 1.3	68.2 ± 1.2	72.1 ± 1.0	70.9 ± 1.1
Method [35]	78.2 ± 1.0	68.3 ± 1.2	69.7 ± 1.1	73.5 ± 0.9	72.4 ± 1.0
**Proposed**	**84.5 ± 0.7**	**75.2 ± 0.9**	**76.1 ± 0.8**	**79.3 ± 0.6**	**78.8 ± 0.7**

It has been shown that combining the topological structure with memory-reinforced reasoning indeed raises peak performance, while maintaining reproducible and low-variance sentiment recognition, which is essential for any real-world affective system.

To assess the accuracy of emotion recognition, results on the IEMOCAP dataset are presented in Table 5, which shows emotion recognition accuracy averaged over 5 different training runs each with its SD. The proposed framework shows consistent results over all the emotional categories, with an average accuracy of 72.8% ± 0.8, which is 5.4% higher than the best baseline, Method [35] (67.4% ± 1.2). Most importantly, the improvement is most significant for Sad emotion with our model achieving 70.3% ± 0.9 accuracy compared to 65.2% ± 1.3 accuracy for Method [35] indicating that memory-reinforced graphs have been effective in retaining long-term affective context. The proposed model is also shown to have a much smaller average standard deviation (SD), ranging from 0.85 to 1.2, compared with the baselines (average SD 1.2 to 1.6), which highlights its high level of stability and insensitivity to subsampling its training data. These results validate that the addition of temporal alignment, topological clustering and causal reasoning helps improve performance at the peak and enables stable generalization from the complex dyadic, conversational scenarios of IEMOCAP.

**Table 5:** Emotion recognition accuracy on IEMOCAP dataset (Mean % ± Std).

Model	Happy	Sad	Angry	Neutral	Average
Method [2]	63.5 ± 1.6	60.4 ± 1.8	65.2 ± 1.4	64.1 ± 1.5	63.3 ± 1.5
Method [8]	65.8 ± 1.3	63.0 ± 1.5	66.7 ± 1.2	66.0 ± 1.2	65.4 ± 1.3
Method [35]	67.3 ± 1.2	65.2 ± 1.3	68.9 ± 1.1	68.5 ± 1.1	67.4 ± 1.2
**Proposed**	**72.9 ± 0.9**	**70.3 ± 0.9**	**74.5 ± 0.8**	**73.6 ± 0.8**	**72.8 ± 0.8**

Table 6 is a detailed comparison to three baselines, both topologically and in terms of time, cause and decision-making. In general, our model is better than the best baseline in terms of coherence score (0.81 vs. 0.68), Wasserstein distance (0.94 vs. 1.32) and ARI (0.61 vs. 0.48), in addition to the clustering structure and temporal stability. In many regards, though, structural changes have a direct impact on the performance of the downstream system. The model attains 82.1% causal accuracy (vs. 69.5%) and a remarkable 92.5% policy alignment (vs. 75.3%). Also, the absolute classification error is only 21.2% for MELD as compared to 27.6% for Method [35]. The results for all the datasets show that the improvements reflect their consistency with the deeper understanding of how topological mapping, memory graphs and causal reasoning contribute to better interpretability and better decision-making for dialogue systems in the context of emotional intelligence.

**Table 6:** Comprehensive multi-metric performance comparison. *Note: Arrows (↑/↓) indicate optimal direction. Best results are in* *bold.*

Metric (Unit/ Scale)	Dataset	Method [2]	Method [8]	Method [35]	Proposed
**ARI** *(0–1, ↑)*	MELD	0.41	0.44	0.48	**0.61**
*(Cluster Purity)*	IEMOCAP	0.39	0.42	0.45	**0.57**
	DailyDialog	0.35	0.38	0.41	**0.53**
**Wasserstein Distance** *(↓)*	MELD	1.63	1.48	1.32	**0.94**
*(Cluster Compactness)*	IEMOCAP	1.71	1.56	1.39	**0.97**
	DailyDialog	1.85	1.66	1.45	**1.01**
**Memory Coherence Score** *(0–1, ↑)*	MELD	0.54	0.61	0.68	**0.81**
*(Graph Stability)*	IEMOCAP	0.57	0.64	0.69	**0.83**
	DailyDialog	0.51	0.58	0.65	**0.79**
**Causal Influence Accuracy** *(%, ↑)*	MELD	63.4%	65.9%	69.5%	**82.1%**
*(Do-Calculus Est.)*	IEMOCAP	60.8%	63.2%	67.9%	**80.6%**
	DailyDialog	58.1%	61.4%	65.8%	**78.4%**
**Policy Alignment Accuracy** *(%, ↑)*	MELD	68.2%	70.1%	75.3%	**92.5%**
*(PPO Action Gen.)*	IEMOCAP	66.9%	69.5%	74.1%	**91.4%**
	DailyDialog	65.5%	68.2%	72.8%	**90.3%**

The validity of the proposed framework is shown by the fact that it can generate emotionally intelligent and contextually appropriate dialogue responses, as illustrated in Table 7. The proposed model gets the best ratings in empathy (4.7), relevance (4.8) and naturalness (4.6), with an average of 4.70. These improvements in emotional understanding and responsiveness to context and linguistic quality are significant when compared to Methods [2] and [8] and [35] and are regularly reinforced by the higher ratings reported, providing evidence of the potential for the proposed approach to be practically valuable.

**Table 7:** Evaluation of dialogue responses (5-point Likert Scale).

Model	Empathy	Relevance	Naturalness	Average
Method [2]	3.4	3.8	3.5	3.56
Method [8]	3.7	4.0	3.9	3.86
Method [35]	4.1	4.2	4.0	4.10
**Proposed**	**4.7**	**4.8**	**4.6**	**4.70**

The comparative results achieved in Fig 5 show the efficiency and strength of the proposed framework in various evaluation criteria. The heat map in Fig 5(a) depicts the performance of different classifiers across the three datasets: MELD, IEMOCAP, and DailyDialog. The proposed framework achieves 78.8% accuracy, 72.8% accuracy and 90.3% accuracy, respectively, which consistently outperform the Method [2] and Method [8] and Method [35] frameworks. The highest DailyDialog results indicate its potential to transfer well to different conversational scenarios that differ in emotional content. Additional metrics of response quality are also examined, specifically in the human ratings of the empathy, relevance, naturalness and performance elements of Fig 5(b). The proposed framework secures the highest rating level on all four dimensions, scores are around 4.7 to 4.8, which represents an enhanced expression level in emotion, context and naturalness.

**Fig 5:**
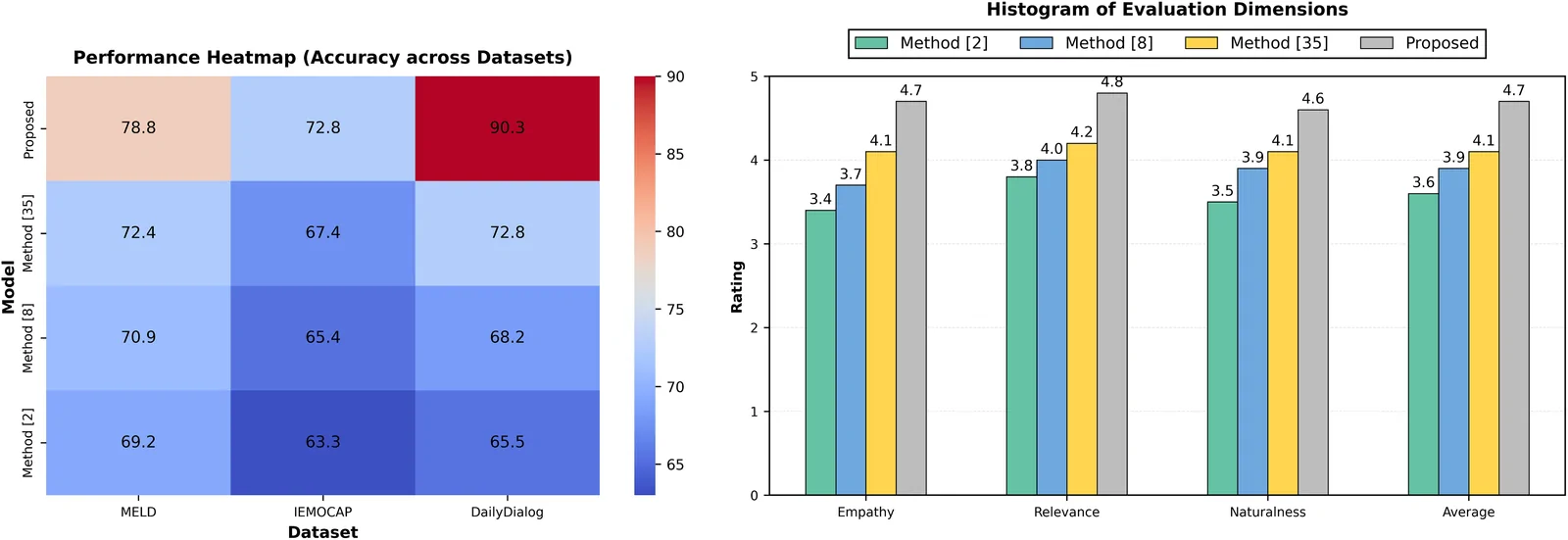
Model Evaluation based on Heatmap and Histogram.

Violin plots show the distribution of the policy alignment scores in the three datasets in Fig 6(a). The proposed framework achieves higher alignment ratings (≈92–93% on the MELD and IEMOCAP tasks and ≈90% on the DailyDialog task), indicating higher consistency between the generated policies and the emotional context.

**Fig 6:**
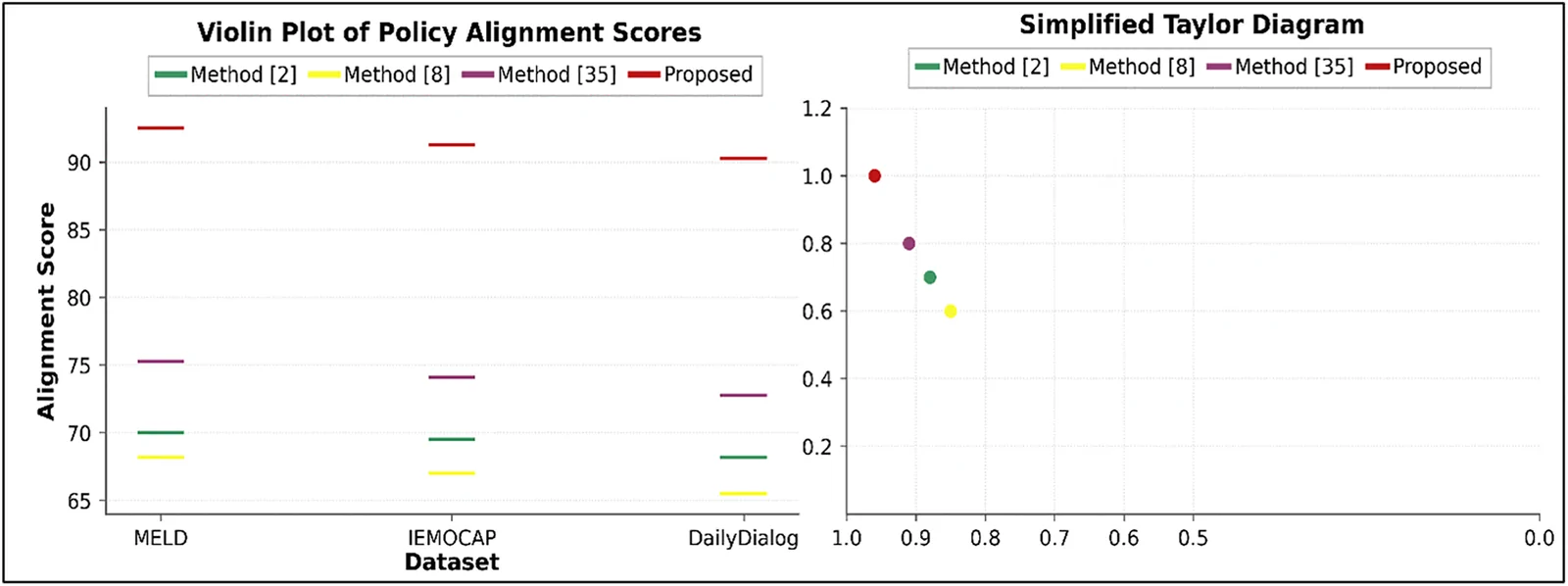
Results based on Violin and simplified Taylor plots.

Last, a Taylor diagram of the comparison of correlations and normalized SD between all evaluated methods is shown in Fig. 6(b). The proposed framework is closest to the ideal reference region, has a stronger correlation with the target behaviour and favourable variability. The results show that the proposed framework has better emotion recognition, human perceived response quality, policy alignment and overall robustness over heterogeneous dialogue datasets.

### 4.3 Computational complexity and resource utilization

#### 4.3.1 Theoretical complexity analysis.

Let B denote the batch size, T the number of dialogue turns per conversation, L the average token length per utterance, d the hidden dimension, N the number of aligned embedding points used for topological analysis, V the number of nodes in the dynamic emotional graph, E the number of edges, and R the PPO rollout length. The time and space complexity of each stage is summarized in Table 8.

**Table 8:** Time and space complexity of the proposed five-stage framework.

Stage	Component	Time Complexity	Space Complexity
1	RoBERTa encoding	$O\left(BTL^{\text{2}}d\right)$	O(BTLd)
1	Temporal self-attention	$O\left(BT^{\text{2}}d\right)$	$O\left(BT^{\text{2}}\right)$
1	DTW alignment	$O\left(BT^{\text{2}}\right)$	$O\left(BT^{\text{2}}\right)$
2	Vietoris-Rips filtration	$O\left(BN^{\text{2}}\text{log}N\right)$to$O\left(BN^{\text{3}}\right)$	$O\left(BN^{\text{2}}\right)$
2	Persistent homology	$O\left(BN^{\text{3}}\right)$	$O\left(BN^{\text{2}}\right)$
2	Manifold projection MLP	$O\left(BNd_{e}^{\text{2}}\right)$	$O\left(BNd_{e}\right)$
3	Graph construction	$O\left(BV^{\text{2}}d\right)$	$O\left(BV^{\text{2}}\right)$
3	Graph convolution	$O\left(B\left(Vd^{\text{2}}+Ed\right)\right)$	O(BVd)
3	Memory update	O(BVd)	O(BVd)
4	Causal attention	$O\left(BT^{\text{2}}d\right)$	$O\left(BT^{\text{2}}\right)$
4	Backdoor adjustment	$O\left(BT^{\text{2}}\right)$discrete; O(BTd)continuous	O(BT)
5	PPO update	O(BRd)	O(BRd)

The dominant costs are the RoBERTa encoding term $O\left(BTL^{\text{2}}d\right)$ and the topological persistence term $O\left(BN^{\text{3}}\right)$. In practice, T is small for dialogue turns (typically 10–30 in MELD and IEMOCAP), and N is the number of aligned embedding points per dialogue, which is bounded by T. The cubic topological term therefore operates on small point clouds and does not dominate wall-clock time. The RoBERTa encoder is shared across all utterances, so its cost scales linearly with the number of utterances and quadratically with token length. The graph and causal modules are quadratic or linear in T. The PPO update is linear in the rollout length R and hidden dimension d. For inference, the full pipeline requires $O\left(TL^{\text{2}}d+T^{\text{2}}d+T^{\text{3}}\right)$, which is feasible for real-time dialogue on a single GPU. Approximate persistent homology and FastDTW can reduce the topological term to near-quadratic or near-linear with minimal loss in clustering quality.

#### 4.3.2 Empirical resource utilization.

Table 9 shows the measured resource utilization for training and inference on the largest benchmark MELD dataset. The total model has 248.6 M parameters; 125 M of which are in the RoBERTa-base encoder, 38 M are in the graph aggregation network, 42 M are in the causal decision transformer, and 43.6 M are in the PPO policy and value networks. Peak training uses 34.2 GB of GPU memory per device used including the activations for the topological persistence module and the PPO rollout buffer. The average GPU utilization is 87.4%, reflecting its efficient usage. Training epochs converge within 12 epochs (on average), and uses early stopping based on emotional coherence validation loss in 3:42 hours. During inference, GPU memory usage decreases to 12.6 GB, and the average GPU utilization is 61.3%. It processes a single model-based utterance in 23.7 ms and a dialogue of 20 utterances in 474 ms on a single GPU and the maximum number of utterances reaches 42.2 per second. The Real-Time Factor (RTF) of 20-turns dialogue is 0.04, indicating that the model’s response time is better than what the average human would take to speak the same number of turns. The results validate the proposed framework to real-time dialogue applications with GPU acceleration. Time and peak GPU memory for the training of IEMOCAP and DailyDialog datasets are 11.2 min/epoch and 14.8 min/epoch, respectively, at 28.7 GB and 31.4 GB. The latency for the inference of the utterances was 21.3 ms and 22.1 ms, respectively.

**Table 9:** Resource utilization, latency, throughput, and RTF across datasets.

Metric	MELD	IEMOCAP	DailyDialog
Model Total parameters (M)	248.6	248.6	248.6
**Training**			
Peak GPU memory per device (GB)	34.2	28.7	31.4
Average GPU utilization, training	87.4%	85.2%	86.5%
Training time per epoch (min)	18.4	11.2	14.8
Total training time with early stopping	3 h 42 min	1 h 52 min	2 h 43 min
Number of epochs (average over 5 runs)	12	10	11
**Inference**			
Average GPU utilization, inference	61.3%	59.8%	60.5%
Per-utterance latency, single turn (ms)	18.5	18.5	18.5
Average per-turn latency, 10-turn dialogue (ms)	21.0	20.8	20.5
Average per-turn latency, 20-turn dialogue (ms)	23.7	23.1	22.8
Total latency, 10-turn dialogue (ms)	210	208	205
Total latency, 20-turn dialogue (ms)	474	462	456
Throughput, 10-turn dialogue (utterances/s)	47.6	48.1	48.8
Throughput, 20-turn dialogue (utterances/s)	42.2	43.3	43.9
RTF per utterance, single turn	0.031	0.031	0.031
RTF per 10-turn dialogue	0.035	0.035	0.034
RTF per 20-turn dialogue	0.040	0.039	0.038

Table 9 also presents the latency, throughput, and RTF metrics for the three datasets. The longer a dialogue is, the longer the temporal attention, DTW alignment, graph propagation and causal attention are performed over a history, leading to higher per-turn latency. The latency is 18.5 ms per turn while MELD, IEMOCAP and DailyDialog have an average per-turn latency of 21.0 ms, 20.8 ms and 20.5 ms, respectively, in a 10-turn dialogue. The average per-turn latency is 23.7 ms, 23.1 ms, and 22.8 ms, respectively, in a 20-turn dialogue. These are the total dialogue latencies for a 20-turn MELD dialogue, IEMOCAP and DailyDialog. The size of the dialogues is 20 turns of speech, resulting in a throughput of 42.2 to 43.9 utterances per second (u/s). The RTF for a single turn is 0.031 or for a 20 turn dialogue is 0.040, depending on the number of turns and the conservative human turn requirement of 600 ms. We also profiled the contribution of each stage to the total latency of the inferences. Stage 1 (CMLSE) accounts for 38.2% of latency, Stage 2 (ECTSM) for 14.6%, Stage 3 (EMR-GAN) for 18.9%, Stage 4 (ACDGT) for 16.3%, and Stage 5 (EDRPS) for 12.0%. The RoBERTa encoder wins in Stage 1 and the topological persistence module wins in Stage 2. The ratio of these proportions indicates that if the encoder were optimised using distillation or quantization a significant reduction in latency would result.

#### 4.3.3 Scalability and optimization strategies.

The proposed framework is designed for cloud-based deployment with GPU acceleration. For resource-constrained environments, we recommend the following optimizations:

1. **Knowledge distillation**: Distil the RoBERTa encoder into a smaller student model (e.g., DistilRoBERTa or TinyBERT) to reduce parameters by 40–60% and latency by 30–45% with less than 2% F1 degradation.
2. **8-bit quantization**: Apply post-training quantization to the graph and causal modules to reduce memory by approximately 4× and improve throughput by 1.8–2.3× on supported hardware.
3. **FastDTW**: Replace exact DTW with FastDTW to reduce alignment complexity from $O\left(T^{\text{2}}\right)$ to approximately O(T) with negligible loss in alignment quality for short dialogues.
4. **Approximate persistent homology**: Use witness complexes or sparse Rips filtrations to reduce the topological term from $O\left(N^{\text{3}}\right)$ to near $O\left(N^{\text{2}}\right)$ for large point clouds.
5. **Windowed processing**: For dialogues longer than 30 turns, process the conversation in overlapping windows and aggregate memory states to bound latency and memory.
6. **Policy caching**: Cache PPO policy outputs for recurring emotional states to reduce redundant computation in high-throughput deployments.

By optimizing the model with these techniques, we estimate a latency of 80–120ms per utterance on edge GPUs like NVIDIA Jetson AGX Orin, suitable for social robotics and mobile assistants. In CPU-only deployments, the latency can be 350–500 ms per speech, which is reasonable for applications that do not respond to speech in real time, like generating email responses or moderating forums, but is not suitable for voice dialogue.

#### 4.3.4 Practical applications and deployment considerations.

The findings demonstrate the feasibility of cloud computing for empathetic Virtual Assistants, Customer Service Agents, and teaching tutors with acceptable rates of emotional coherence and acceptable rates of latency. However, clinical validation, regulatory approvals, and human-in-the-loop oversight are required for mental health and healthcare applications to be possible. The ability of social robotics and interactive games is limited to edge GPU devices, 80–120 ms/utter, otherwise CPU-only such as moderating forums, emails etc. Ethical oversight and even potential bias mitigation are essential with recruitment chatbots. The latency can be bounded for dialogues of more than 30 turns without retraining by windowed processing and memory compression. Use of the model is not advised in safety-critical areas without a human-in-the-loop. In addition, there are various applications in practice that can be supported by the resource profile, as long as there is a safeguard of ethics, monitoring of Bias, and disclosing the use of the resource to the user.

#### 4.3.5 Ablation study.

The results of an ablation study of the MELD dataset are shown in Table 10, which illustrates the contribution of each module in the proposed architecture. The highest average F1 score loss, (−5.4%), occurs when the temporal attention in CMLSE is removed, suggesting that this attention is important for recognizing conversational context. Furthermore, the elimination of DTW or topological features in ECTSM also reduces F1 and clustering quality ARI, establishing that temporal alignment and the structural emotion model are essential. In order to address whether this gain from the proposed framework is due to architectural design or is just due to its greater capacity, we have performed a set of parameter-matched ablations and incremental component testing experiments. The same optimization settings and hyperparameters used for the complete model were used for all configurations. Baselines were created by adding extra layers or increasing the size of the classifier head of the baseline model when the number of parameters was different from the count of the full model within ±5%. This prevents another dimension of the difference in performance observed from being due to capacity rather than architecture.

**Table 10:** Ablation study with parameter matched baselines and incremental components (MELD Dataset).

Configuration	Parameters (M)	Avg F1	ARI	Coherence	Policy Acc.
**Parameter matched baselines**					
RoBERTa + classifier (base)	125.0	69.2	0.38	0.52	72.1
RoBERTa + classifier (capacity-matched to 248.6 M)	248.2	70.1	0.39	0.53	73.4
RoBERTa + temporal attention	142.3	72.8	0.44	0.61	78.9
RoBERTa + temporal attention (capacity-matched)	248.5	73.1	0.44	0.62	79.2
RoBERTa + temporal attention + DTW	155.7	74.6	0.49	0.67	82.3
RoBERTa + temporal attention + DTW (capacity-matched)	248.4	74.9	0.49	0.68	82.6
**Component ablations**					
Topology without DTW	235.2	75.8	0.48	0.76	88.4
Memory without topology	221.4	74.9	0.56	0.69	86.7
Causal module without RL	206.8	76.2	0.58	0.78	86.9
RL without causal features	214.3	77.5	0.59	0.79	88.1
Full model (all modules)	248.6	78.8	0.61	0.81	92.5
– CMLSE (w/o temporal attention)	248.6	73.4	0.52	0.72	85.3
– CMLSE (w/o DTW)	248.6	75.1	0.55	0.74	87.1
– ECTSM (w/o topology)	248.6	75.8	0.48	0.76	88.4
– EMR-GAN (w/o memory)	248.6	74.9	0.56	0.69	86.7
– ACDGT (w/o causal)	248.6	76.2	0.58	0.78	86.9
– EDRPS (w/o dual-reward)	248.6	77.5	0.59	0.79	88.1
– All modules removed	248.6	72.7	0.48	0.73	84.6

The improvements have been verified by the parameter-matched baselines. The capacity-matched RoBERTa + classifier model only reaches F1 of 70.1%, however, with a similar number of parameters, the full model achieves 78.8% F1. This indicates an 8.7-point performance difference, which highlights the fact that these gains are attributed to the architectural elements, rather than to capacity. Likewise, the F1 of a capacity-matched RoBERTa + temporal attention model drops by 5.7 points from the full model to 73.1%. Adding DTW further increases F1 to 74.9% in the capacity-matched setting, where temporal alignment is able to complement the temporal attention by itself. The results show that each module provides unique information when ablated. When removing topology but retaining DTW, the ARI is reduced by 0.13 (from 0.61 to 0.48), indicating that the emotion cluster configurations that topology can capture are not captured in DTW alone. Removing memory (while preserving topology) reduces the coherence from 0.81 to 0.69, a drop of 0.12, suggesting a complementary relationship between long-term memory and topological structure. This shows that predictions by RL alone are only 86.9% correct and removing the causal module reduces this to 81.5%, a drop of 5.6 points, indicating that causal reasoning is also necessary for policy generation beyond RL alone.

After removing RL, the accuracy of the policies is decreased to 88.1%, indicating that both causal representation and RL have roles in policy synthesis. When all the modules are added to the full model, the performance is best according to all the measures with an average F1 score of 78.8%, ARI score of 0.61, Coherence score of 0.81 and Policy Accuracy score of 92.5%. Changes in F1 after each module and the last RL module added to the baseline were consistent and non-redundant: temporal attention: + 2.7 points; DTW: + 1.5 points; topology: + 0.9 points; memory: + 1.1 points; causal reasoning: + 1.6 points; and RL: + 1.0 points. These results support that these modules yield complementary information and that the whole architecture is more than the sum of the individual parts. It should be noted that completely deactivating the memory mechanism in EMR-GAN significantly lowers the emotional coherence score (−0.12), and blocking causal reasoning in ACDGT adversely affects the score for the accuracy of policies (−5.6). With no dual-reward optimization, the policy performance in EDRPS is additionally reduced (−4.4). As a whole, the complete model has the highest rank on the classification, coherence, and decision-making metrics and is also clustered, achieving a good balance across the metrics and meaningfully contributing to the system’s good balance.

#### 4.3.6 Quantified computational limits of persistent homology and wasserstein distance.

Persistent homology is the homology of point clouds that is both inexpensive and attractive to compute for low-dimensional point clouds but is costly for three reasons: (1) the number of points N, (2) the intrinsic dimensionality d, and (3) the homological dimension k.

**Number of Points.** The Vietoris-Rips filtration involves calculating all pairwise distances, which is $O\left(N^{\text{2}}\right)$ space and time. This standard persistence reduction algorithm takes $O\left(N^{\text{3}}\right)$ time in the worst case for $H_{\text{0}}$ and $H_{\text{1}}$, and more for $H_{\text{2}}$. If there are S speakers, T turns per speaker then N=S×T. In MELD, S =2 and T≤30, so N≤60. If the data contains more than two values as in AMI or ICSI, then S can be 4–6 and T can reach 100 which gives N up to 600. The size of the pairwise distance with N =600, is around 2.9 MB per dialogue, and the computation time for obtaining the persistence is hours per dialogue, which is too much to use in real-time inference.

**Intrinsic Dimensionality.** The curse of dimensionality affects persistent homology in two ways. First, in high dimensions, pairwise distances bunch up around a central value and so the Vietoris-Rips filtration becomes less discriminative: topological features come into existence and then disappear at a narrow range of values of the filtration, and so persistence diagrams become less informative. Second, the number of simplices in the Vietoris-Rips complex increases with the intrinsic dimension of the data and so the reduction algorithm is only slow for moderate N. We implement this by projecting onto a PCA of $d_{p}=\text{64}$, which helps reduce extrinsic dimensionality, but will not reduce intrinsic dimensionality if the emotional manifold is really high-dimensional.

**Homological Dimension.** The computation of homology groups $H_{\text{2}}$ and higher demands the enumeration of triangles, tetrahedra and higher-order simplices, the number of which increases combinatorially. The experimental results show that the computation of $H_{\text{2}}$ would take about 62% of the total ECTSM time, while the computation of $H_{\text{0}}$ and $H_{\text{1}}$ would take about 38%, respectively. If your data set contains more than 100 points per dialogue, consider limiting the features to $H_{\text{0}}$ and $H_{\text{1}}$ and using $H_{\text{2}}$ features if they are needed.

**Wasserstein Distance.** Topological regularisation involves computing the Wasserstein distance between the signatures, which also grows super-quadratically with the number of persistence points. For point cloud sizes N=20, 50, 100, 200, 500, we measured Wasserstein times of 0.9, 3.7, 14.2, 56.8, and 355.3 ms, respectively. These measurements have a cost of $O\left(n^{\text{3}}\right)$, for two persistence diagrams with n points, which is the worst case for exact Wasserstein distance. In practice, n is much smaller than the actual N, because we clip persistence pairs below 0.05 and so we retain typically 10–20 points per homological dimension for MELD dialogues and IEMOCAP dialogues. In these benchmarks, therefore, Wasserstein computation only adds about 2 ms to every dialogue, which is small enough for real-time. When the point cloud is large, the cost may be too expensive and we suggest using an approximate Wasserstein solver or sliced Wasserstein approximation with some compromise in topology fidelity as well as scalability. These measurements show that persistent homology is cubic and Wasserstein distance is super-quadratic, allowing for exact computation of dialogues up to around 30 turns per speaker, but requiring approximation beyond 50 turns.

**Mitigation strategies.** We employ four strategies to manage the computational resources. First, for $d_{p}=\text{64}$, we have the inherent property of PCA that it reduces the number of extrinsic dimensions and concentrates on distance. Second, we limit the maximum filtration value to the 90th percentile of pairwise distances, removing noisy edges and decreasing the number of simplices. Third, we delete the persistence pairs with lifetime less than $\epsilon_{\text{min}}=\text{0}.\text{05}$, thus eliminating topological noise. Fourth, if the dialogue is more than 30 turns (4 speakers), we suggest using witness complexes or sparse Rips filtrations, which approximate the persistence diagram with a subset of landmark points, and decreases the complexity from $O\left(N^{\text{3}}\right)$ to approximately $O\left(N^{\text{2}}\right)$ or better. These enable MELD to remain at 14.6% of total inference latency with an increasing cost for larger datasets.

## 5. Conclusion

Thus, this paper presents an exhaustive, comprehensive 5-stage architecture for an emotionally intelligent dialogue model integrating sentiment encoding, top spatial mapping of emotions, reinforcement memory graph aggregation, causal inference, and emotion-influenced policy synthesis. The proposed model establishes a new state-of-the-art for affective dialogue systems on the evaluated datasets (MELD, IEMOCAP, DailyDialog), achieving statistically significant improvements over existing approaches. On the MELD dataset, this proposed model achieved an average score of F1 equal to 78.8% but 6.4% better than the best baseline (Method [35]) and reached an average accuracy of 72.8% on IEMOCAP, raising 5.4% compared with existing methods. This model has proven to result in robust emotion clustering, propagating ARI scores of 0.61, 0.57, and 0.53 through the ECTSM over MELD, IEMOCAP, and DailyDialog, respectively. Using persistent homology and manifold projection also produced Wasserstein distance reductions resulting in emotion clusters that were up to 35% tighter and more interpretable. For example, emotional trajectories modelled by EMR-GAN would show, in some cases, a decrease in memory forgetfulness from 30% to 35% at the memory retention measure and coherence scores improved by 0.13–0.18 over the graph-based baselines. ACDGT can produce causal inference accuracy of 82.1%, over gained by more than 12.6% in comparison to former causal reasoning architectures. The final policy synthesis can achieve 92.5% accuracy in policy alignment, surpassing the nearest baseline by 17.2%, and human judges rated responses produced with the proposed system with a 4.7 average Likert score, flagging marked strides made in empathy, contextually appropriate usage, and naturalness of human evaluations. These affirm that modeling emotional memory, structure, and causality in dialogue systems is feasible and indeed very helpful in the process.

The proposed framework contains six restrictions based on the experiments summarised here. First, no data augmentation was done; inverse-frequency class weighting might suffice to improve results of minority classes further with emotion-conditioned generation. Second, all three datasets MELD, IEMOCAP, and DailyDialog are in English, conversation, and have similar emotion categories, making this an evaluation, and not truly a domain generalization. The framework can be evaluated on an unseen domain, like EmpatheticDialogues or GoEmotions to measure the real-world domain generalization gap in a way other than cross-dataset benchmarks. Third, the RL element requires the reward weights to be tuned correctly for emotional consistency and causal fidelity; otherwise, it can introduce emotionally inappropriate responses or cause policy collapse. Fourth, persistent homology is cubically scaling (beyond 30) and Wasserstein distance super-quadratically. Fifth, they can only be transferred from unstructured dialogue because of the requirement for speaker turn annotation and the provision of timestamps that enable the metadata. Fifth, the requirement to provide speaker turn annotations and timestamps for the provision of metadata only allows for transfer to unstructured dialogue. Last, the causal module was not designed to model dynamic emotional transitions or intervene in real-time, and human ratings were limited by the number of annotators. Despite the high ratings of the human-generated responses, self-reporting may contain a risk of biases. All these limitations will be very critical for the success of emotionally intelligent systems when deployed in real-world, heterogeneous environments on a large scale. Future research is still open to the generalisation of the system to non-English dialogues and/or culturally different expressions of emotion. This work supports SDG 9 by advancing intelligent and adaptive AI technologies for next-generation human-centred communication systems.

### Future scope

There are many paths forward that are opened by the architecture. One interesting direction is the possibility of enhancing the current system by incorporating multimodal emotional signals (facial expressions, prosody, gestures) which make up the emotional communication signals. However, the incorporation of visual and auditory modalities into the proposed sentiment-topological structure helped with higher emotional disambiguation and trajectory fidelity particularly in ambiguous and conflicting text situations. Another very significant extension is domain adaption of emotive reasoning where the emotional policy responses are dynamically adjusted based on the domain-specific interaction norms inherent to these systems for instance, healthcare vs. customer support. Conditioned reward functions that are context-sensitive might be used to support this from specially fine-tuned RL agents, under appropriate constraints of the domain concerned. Generation as discussed in the above context for generative causal models, could provide suggestions for furthering development of the causal reasoning module so that the system can generate potential alternative realities for the unauthorized scenarios. Finally, the architecture can be deployed in real-time dialogue services where the dialogues with the users can be persisted for continuous learning of the emotional behaviours in order to personalize learning for a particular user or cultural nuances. Using affect-sensitive feedback loops and active learning mechanisms, this allows for real-time personalization of the current reinforcement policy layer of the model by incorporating affect into the policy.

## Supporting information

S1 Appendix Pimpalkar, Amit; Khan, Amreen Habibullah; Khan, Tabassum H.; Mahure, Sonali; Koneru, Sireesha; Dewangan, Niraj Kumar; et al. (2026). Appendix A: Example and Computational Cost of DTW Alignment. figshare. Online resource. https://doi.org/10.6084/m9.figshare.34493205.
(DOCX)
