# Supplementary material for: A multi-stage framework for emotionally intelligent dialogue systems using topological sentiment encoding and causal policy optimization
Source: PLoS One. 2026 Oct 28;21(10):e0361365. doi: 10.1371/journal.pone.0361365 (PMC13716236; doi:10.1371/journal.pone.0361365)
Supplement: Supplementary file 1 [file pone.0361365.s001.docx]

**Appendix A: Example and Computational Cost of DTW Alignment**

A detailed illustration of the DTW alignment procedure described in Section 3.1.3 is given in this appendix which explicitly walks through Equations (9) through (14). It also presents wall-clock statistics of exact DTW and FastDTW on the MELD dataset. The main text has been abridged with a summary and the reference back to this appendix for operational details.

**A.1 Worked Example of DTW Alignment**

We use a dialogue excerpt of the MELD dataset that contains two speakers (Speaker A and Speaker S) and six dialogue turns. Speaker A says: "Joy" in turns 1 and 5, and "Neutral" in turn 3. Speaker S answers Neutral (T2), Surprise (T4) and Neutral (T6). To make it easier to understand, simplified four-dimensional projections of the RoBERTa embeddings are used, whereas the 768 dimensional versions of the embeddings are used in the actual model.

**Equation (9)** defines the two sequences to be aligned:

$$\boldsymbol{H}_{A}=\left( \tilde{h}^{\left( A , 1 \right)} , \tilde{h}^{\left( A , 3 \right)} , \tilde{h}^{\left( A , 5 \right)} \right), \boldsymbol{H}_{S}=\left( \tilde{h}^{\left( B , 2 \right)} , \tilde{h}^{\left( B , 4 \right)} , \tilde{h}^{\left( B , 6 \right)} \right)$$

The contextualized embeddings, simplified to four dimensions, are:

| **Utterance** | **Speaker** | **Emotion** | **Embedding** |
| --- | --- | --- | --- |
| $u^{\left( A , 1 \right)}$ | A | Joy | $\tilde{h}^{\left( A , 1 \right)}=\left[ 0.90 , \text{ } , 0.10 , \text{ }0.10\text{ }0.10 \right]$ |
| $u^{\left( A , 3 \right)}$ | A | Neutral | $\tilde{h}^{\left( A , 3 \right)}=\left[ 0.30 , \text{ } , 0.30 , \text{ }0.70\text{ }0.30 \right]$ |
| $u^{\left( A , 5 \right)}$ | A | Joy | $\tilde{h}^{\left( A , 5 \right)}=\left[ 0.80 , \text{ } , 0.20 , \text{ }0.20\text{ }0.20 \right]$ |
| $u^{\left( S , 2 \right)}$ | S | Neutral | $\tilde{h}^{\left( S , 2 \right)}=\left[ 0.30 , \text{ } , 0.30 , \text{ }0.70\text{ }0.30 \right]$ |
| $u^{\left( S , 4 \right)}$ | S | Surprise | $\tilde{h}^{\left( S , 4 \right)}=\left[ 0.70 , \text{ } , 0.70 , \text{ }0.10\text{ }0.50 \right]$ |
| $u^{\left( S , 6 \right)}$ | S | Neutral | $\tilde{h}^{\left( S , 6 \right)}=\left[ 0.30 , \text{ } , 0.30 , \text{ }0.70\text{ }0.30 \right]$ |

**Equation (10)** defines the local alignment cost as the squared Euclidean distance between embedding pairs:

$$C\left( m , n \right)=\left\| \tilde{h}^{\left( A , m \right)}-\tilde{h}^{\left( S , n \right)} \right\|_{2}^{2}$$

Evaluating this for all $m,n\in\left\{ 1 , 2 , 3 \right\}$gives the local cost matrix:

|  | $S_{1}$**(Neutral)** | $S_{2}$**(Surprise)** | $S_{3}$**(Neutral)** |
| --- | --- | --- | --- |
| $\boldsymbol{A}_{\boldsymbol{1}}$**(Joy)** | 0.80 | 0.56 | 0.80 |
| $\boldsymbol{A}_{\boldsymbol{2}}$**(Neutral)** | 0.00 | 0.72 | 0.00 |
| $\boldsymbol{A}_{\boldsymbol{3}}$**(Joy)** | 0.52 | 0.36 | 0.52 |

For example, $C\left( 1 , 2 \right)=\left( 0.90-0.70 \right)^{2}+\left( 0.10-0.70 \right)^{2}+\left( 0.10-0.10 \right)^{2}+\left( 0.10-0.50 \right)^{2}=0.04+0.36+0.00+0.16=0.56$. The low cost at $C\left( 1 , 2 \right)$ indicates that Speaker A’s Joy embedding is close to Speaker $S$’s Surprise embedding, reflecting the emotional escalation between the two turns.

**Equation (11)** defines the cumulative DTW cost recursively:

$$D\left( m , n \right)=C\left( m , n \right)+\min\left\{ D , \left( m , -1n \right) , \text{ }D\left( m , n-1 \right)\text{ }D\left( m , -1n-1 \right) \right\}$$

with boundary initialization $D\left( 1 , 1 \right)=C\left( 1 , 1 \right)$. Computing row by row yields:

|  | $S_{1}$ | $S_{2}$ | $S_{3}$ |
| --- | --- | --- | --- |
| $A_{1}$ | $D\left( 1 , 1 \right)=0.80$ | $D\left( 1 , 2 \right)=0.56+0.80=1.36$ | $D\left( 1 , 3 \right)=0.80+1.36=2.16$ |
| $A_{2}$ | $D\left( 2 , 1 \right)=0.00+0.80=0.80$ | $D\left( 2 , 2 \right)=0.72+\min\left( 0.80 , 1.36 , 0.80 \right)=1.52$ | $D\left( 2 , 3 \right)=0.00+\min\left( 1.36 , 2.16 , 1.52 \right)=1.36$ |
| $A_{3}$ | $D\left( 3 , 1 \right)=0.52+0.80=1.32$ | $D\left( 3 , 2 \right)=0.36+\min\left( 0.80 , 1.52 , 1.32 \right)=1.16$ | $D\left( 3 , 3 \right)=0.52+\min\left( 1.52 , 1.36 , 1.16 \right)=1.68$ |

The minimum cumulative cost is $D\left( 3 , 3 \right)=1.68$, representing the total alignment cost between the two speakers’ emotional trajectories.

**Equation (12)** obtains the optimal alignment path by backtracking from $\left( T_{A} , T_{S} \right)=\left( 3 , 3 \right)$. At each step, we move to the predecessor that achieved the minimum in Equation (11):

- At $\left( 3 , 3 \right)$: the minimum of $\left\{ D , \left( 2 , 2 \right) , =1.52\text{ }D\left( 2 , 3 \right)=1.36\text{ }D\left( 3 , 2 \right)=1.16 \right\}$ is $1.16$ at $\left( 3 , 2 \right)$. So $\left( 3 , 3 \right)\leftarrow\left( 3 , 2 \right)$.
- At $\left( 3 , 2 \right)$: the minimum of $\left\{ D , \left( 2 , 1 \right) , =0.80\text{ }D\left( 2 , 2 \right)=1.52\text{ }D\left( 3 , 1 \right)=1.32 \right\}$ is $0.80$ at $\left( 2 , 1 \right)$. So $\left( 3 , 2 \right)\leftarrow\left( 2 , 1 \right)$.
- At $\left( 2 , 1 \right)$: the only predecessor is $\left( 1 , 1 \right)$, since $D\left( 2 , 1 \right)=C\left( 2 , 1 \right)+D\left( 1 , 1 \right)$. So $\left( 2 , 1 \right)\leftarrow\left( 1 , 1 \right)$.

Reversing the backtrack, the optimal alignment path is:

$$\mathcal{P}^{*}=\left\{ \left( 1 , 1 \right) , \text{ } , \left( 2 , 1 \right) , \text{ }\left( 3 , 2 \right)\text{ }\left( 3 , 3 \right) \right\}$$

This path is monotonic and satisfies the DTW boundary conditions. It has a clear emotional interpretation:

- $\left( 1 , 1 \right)$: Speaker A’s Joy aligns with Speaker $S$’s Neutral at the start of the exchange.
- $\left( 2 , 1 \right)$: Speaker A’s Neutral also aligns with Speaker $S$’s Neutral, capturing emotional matching between the two speakers.
- $\left( 3 , 2 \right)$: Speaker A’s Joy aligns with Speaker $S$’s Surprise, capturing the emotional escalation.
- $\left( 3 , 3 \right)$: Speaker A’s Joy also aligns with Speaker $S$’s subsequent Neutral, capturing the emotional resolution.

In other words, Speaker $A$'s joy to neutral transition is contained within Speaker $S$'s neutral to neutral transition, and Speaker $S$'s surprise to neutral transition is within Speaker $A$'s joy to neutral transition. This is the type of long-range cross-speaker affective dependencies which is not captured by fixed-window models. Using the aligned representation for each position m, the representation for the position m is computed in **Equation (13)** by aggregating the embeddings of the DTW match positions.

$$\hat{h}^{\left( A , m \right)}=\frac{1}{\mathcal{\mid M}\left( m \right)\mid}\sum_{n\mathcal{\in M}\left( m \right)} \tilde{h}^{\left( S , n \right)}$$

**Equation (14)** defines the match set:

$$\mathcal{M}\left( m \right)=\left\{ n:\left( m , n \right)\in\mathcal{P}^{*} \right\}$$

Applying these to the path $\mathcal{P}^{*}$gives:

- $\mathcal{M}\left( 1 \right)=\left\{ 1 \right\}$, so $\hat{h}^{\left( A , 1 \right)}=\tilde{h}^{\left( S , 2 \right)}=\left[ 0.30 , \text{ } , 0.30 , \text{ }0.70\text{ }0.30 \right]$
- $\mathcal{M}\left( 2 \right)=\left\{ 1 \right\}$, so $\hat{h}^{\left( A , 3 \right)}=\tilde{h}^{\left( S , 2 \right)}=\left[ 0.30 , \text{ } , 0.30 , \text{ }0.70\text{ }0.30 \right]$
- $\mathcal{M}\left( 3 \right)=\left\{ 2 , 3 \right\}$, so $\hat{h}^{\left( A , 5 \right)}=\frac{1}{2}\left( \tilde{h}^{\left( S , 4 \right)}+\tilde{h}^{\left( S , 6 \right)} \right)=\left[ 0.50 , \text{ } , 0.50 , \text{ }0.40\text{ }0.40 \right]$

Each representation of the aligned representations now has cross-speaker emotional context information at each turn. For instance, the blending of embeddings for the Speakers Surprise and Neutral in the mentioned example $\hat{h}^{\left( A , 5 \right)}$ means that Speaker $A$’s last Joy is presented within the context of the emotion unfolding in Speaker $S$’s voice, not standalone. These are then turned into aligned representations and handed over to Stage 2 (ECTSM) for the topological analysis.

**A.2 Computational Cost of DTW Alignment**

The time and space complexity of the standard DTW algorithm is $O\left( T_{i}\times T_{j} \right)$, where $T_{i}$ and $T_{j}$ being the length of the two sequences to be aligned. This is $O\left( T^{2} \right)$ per speaker pair for dialogue with Tturns per speaker. It is reduced with FastDTW to about linear time and space complexity by means of a multilevel strategy that consists of recursively projecting a warp path from a coarser resolution to a finer resolution and refining the path locally. Results of exact DTW and FastDTW on a single A100 GPU for the MELD dataset are listed in **Table A1**. All measurements are averaged over 1,000 dialogues with $T=10$ turns per speaker.

**Table A1: Computational Cost of DTW Alignment on MELD**

| **Method** | **Complexity** | **Avg. Time per Dialogue**  **(ms)** | **Avg. Memory**  **(MB)** | **Alignment Quality**  **(cosine similarity)** |
| --- | --- | --- | --- | --- |
| Exact DTW | $O\left( T^{2} \right)$ | 12.4 | 4.2 | 0.94 |
| FastDTW (radius = 1) | $O\left( T \right)$ | 4.1 | 1.8 | 0.91 |
| FastDTW (radius = 2) | $O\left( T \right)$ | 5.8 | 2.3 | 0.93 |

Average time for Exact DTW is 12.4ms, FastDTW with radius 1 has an average of 4.1ms, a 3.0× speed up. FastDTW with radius 2 takes 5.8 ms, which is 2.1× faster than exact DTW, and achieves a higher quality of alignment. If we take average dialogue length to be about 10 turns in MELD, the quadratic term is not significant even with exact DTW. We recommend the use of FastDTW with radius of 2 (latency reduction) for longer dialogues (more than 30 turns) to preserve the quality of alignment. These measurements confirm the overall inference latency that was reported above in Table 10, which accounts for about 8-12 percent of the per-turn latency. The cost of the DTW module is spread out across all turns; in the full model, one DTW module is used for each speaker pair, for each dialogue. The cost of a single DTW per turn for a 10-turn MELD dialogue is around 1.24 ms or nearly 5.2 per cent of the 23.7 ms per turn latency. The per-turn DTW cost increases to ~2.48 ms after 20 turns in the 20 turn dialogue, or some 10.5 percent of the per-turn latency. This shows that the RoBERTa encoder and the PPO policy head are over 50 percent of the inference time, which is not the key computational bottleneck.

**Implementation Details of ECTSM**

The ECTSM component receives the aligned contextualized representations $\left\{ \hat{h}^{\left( s , t \right)} \right\}$ from Stage 1, and outputs a fixed-dimensional emotional manifold representation for every utterance. This process is carried out in four stages: Point cloud construction, Vietoris–Rips filtration, persistent homology computation, and persistence feature mapping.

**Point cloud construction.** For each dialogue, the aligned embeddings from all speakers and turns are collected into a point cloud $\mathcal{X=}\left\{ \hat{h}^{\left( s , t \right)} \right\}_{s,t}\subset\mathbb{R}^{d_{h}}$, where $d_{h}=768$ is the RoBERTa hidden dimension. To reduce dimensionality and computational cost, we apply principal component analysis (PCA) to project the point cloud to $d_{p}=64$ dimensions, retaining approximately 95% of the variance. This projection is computed per dialogue, not per dataset, so that the topological structure reflects the specific emotional geometry of each conversation. The projected point cloud is denoted $\mathcal{X}_{p}\subset\mathbb{R}^{64}$.

**Vietoris–Rips filtration.** We compute the pairwise Euclidean distance matrix $D_{ij}=\parallel\hat{h}_{i}-\hat{h}_{j}\parallel_{2}$ for all points in $\mathcal{X}_{p}$. Next, the Vietoris-Rips complex is built with the GUDHI library under the following parameters:

Maximum filtration value: The 90th percentile of pairwise distances, avoiding enriching the persistence diagram with noisy edges.

$Maximum homology dimension: k=2$: Captures connected components ($H_{0}$), loops ($H_{1}$), and voids ($H_{2}$). Filtration step: adaptive, step size range 100 to maximum filtration value steps.

The reason for $k=2$ is motivated by the observation that emotional trajectories (transitions) in conversation tend to be one-dimensional (such as a cycle of joy–neutral–joy) and two-dimensional (such as a group of similar emotions such as joy–joy–joy). Escalating dimensionality of homology groups led to a significant increase in the volume of data needed for proper estimation and in preliminary experiments did not enhance clustering performance.

**Persistent homology computation.** We calculate the persistence diagram $D_{k}=\left\{ \left( b_{i} , d_{i} \right) \right\}_{i=1}^{n_{k}}$ for $k\in\left\{ 0 , 1 , 2 \right\}$. Persistent ones are associated with lifetime $d_{i}-b_{i}<\epsilon_{\text{min}}=0.05$ using the standard reduction algorithm implemented in GUDHI and are considered topological noise. This threshold was selected by validation-based grid search over $\left\{ 0.01 , 0.03 , 0.05 , 0.08 , 0.10 \right\}$. Typically, dialogues with 10-20 turns contain around 20 points to remember for the first dimension $n_{0}\leq20$, $n_{1}\leq15$, and $n_{2}\leq8$.

**Persistence feature mapping.** A fixed dimensional vector is obtained by transforming the persistence diagrams through a permutation-invariant Deep Set architecture. The persistence diagrams are then converted to a fixed dimensional vector, using a permutation invariant Deep Set architecture. Each persistence point $\left( b_{i} , d_{i} \right)$ is represented by a shared multilayer perceptron with topologies 2→256→128→128, where the first layer receives birth and death values, and the output from the last layer is a point embedding in 128 dimensions. ReLU activation is applied after every hidden layer, batch normalization is applied after ReLU activation. Sum pooling is used to aggregate the point embeddings and form a 128 dimensional vector for each homological dimension. The three vectors of the dimensions are concatenated into a 384 dimensional vector (3×32), which is then fed into a final MLP with structure 384 →256→128→$d_{e}$, where $d_{e}=64$ is the dimension of the emotional manifold. The final MLP has 2 layers which are dropped with 0.2.

**Training procedure.** The ECTSM module is trained end-to-end with the rest of the framework. The topological regularization term $\lambda_{\text{top}}W_{2}\left( D_{1} , D_{2} \right)$ is computed between the prediction and ground-truth emotion clusters using a grid search over $\left\{ 0.1 , 0.3 , 0.5 , 0.7 , 1.0 \right\}$ with $\lambda_{\text{top}}=0.5$ selected. To calculate gradients through the persistence diagrams, we use the Persistent Homology gradients framework, in which we approximate gradients with respect to input embeddings by spectrally regularizing the boundary operator. The Wasserstein distance is calculated with the auction algorithm, and for the points that are not matched the diagonal matching is considered. From the results in Section 4.3, the ECTSM module accounts for about 14.6% of the total inference latency.

**Hyperparameter summary.** Table A2 summarizes the ECTSM hyperparameters.

**Table A2: ECTSM Hyperparameter Configuration**

| **Parameter** | **Symbol** | **Value** | **Selection Method** |
| --- | --- | --- | --- |
| PCA output dimension | $d_{p}$ | 64 | 95% variance retained |
| Maximum homology dimension | $k$ | 2 | Validation-based selection |
| Maximum filtration percentile | – | 90th | Validation-based selection |
| Minimum persistence threshold | $\epsilon_{\text{min}}$ | 0.05 | Grid search |
| Point encoder hidden units | – | 256, 128 | Validation-based selection |
| Point embedding dimension | – | 128 | Fixed |
| Emotional manifold dimension | $d_{e}$ | 64 | Validation-based selection |
| Wasserstein weight | $\lambda_{\text{top}}$ | 0.5 | Grid search |
| Dropout rate | – | 0.2 | Validation-based selection |
| Batch normalization | – | Yes | Standard practice |
