# Supplementary material for: A multi-stage framework for emotionally intelligent dialogue systems using topological sentiment encoding and causal policy optimization
Source: PLoS One. 2026 Oct 28;21(10):e0361365. doi: 10.1371/journal.pone.0361365 (PMC13716236; doi:10.1371/journal.pone.0361365)
Supplement: Supplementary file 2 [file pone.0361365.s003.docx]

**To,**

**The Editor,**

**PLOS One**

**Thank you for considering our manuscript: “A Multi-Stage Framework for Emotionally Intelligent Dialogue Systems using Topological Sentiment Encoding and Causal Policy Optimization” (Submission ID: PONE-D-26-43909).**

We are delighted to have received a positive evaluation, and we would like to express our appreciation to the Editor, and all reviewers for the critical, constructive positive comments and helpful suggestions in improving the manuscript.

On behalf of all the co-authors, I would like to thank you for inviting us to resubmit our manuscript. The manuscript has been revised in accordance with the comments provided by the Reviewer and Editor. Our detailed, point-by-point responses to the reviewers’ comments are presented below:

We have highlighted the corrected and updated work in cyan color in the revised version of the manuscript.

**Editor Comments**

Dear Dr. Dewangan,

Thank you for submitting your manuscript to PLOS One. After careful consideration, we feel that it has merit but does not fully meet PLOS One’s publication criteria as it currently stands. Therefore, we invite you to submit a revised version of the manuscript that addresses the points raised during the review process.

Please include the following items when submitting your revised manuscript:

- A letter that responds to each point raised by the academic editor and reviewer(s). You should upload this letter as a separate file labeled 'Response to Reviewers'.
- A marked-up copy of your manuscript that highlights changes made to the original version. You should upload this as a separate file labeled 'Revised Manuscript with Track Changes'.
- An unmarked version of your revised paper without tracked changes. You should upload this as a separate file labeled 'Manuscript'.

If you would like to make changes to your financial disclosure, please include your updated statement in your cover letter. Guidelines for resubmitting your figure files are available below the reviewer comments at the end of this letter.

We look forward to receiving your revised manuscript.

Journal Requirements:

When submitting your revision, we need you to address these additional requirements.

1. Please ensure that your manuscript meets PLOS ONE's style requirements, including those for file naming. The PLOS ONE style templates can be found at

<https://journals.plos.org/plosone/s/file?id=wjVg/PLOSOne_formatting_sample_main_body.pdf> and

<https://journals.plos.org/plosone/s/file?id=ba62/PLOSOne_formatting_sample_title_authors_affiliations.pdf>

**Response:**

We thank the editor for this reminder. We have taken the utmost care to follow the PLOS ONE style templates and comply with the journal’s formatting and file-naming requirements. The manuscript structure follows the required order: Title, Authors and Affiliations, Abstract, Introduction, Materials and Methods, Results Discussion, and References. Headings have been standardized, line numbering has been applied to facilitate review, and the reference style has been checked against PLOS ONE guidelines. All uploaded files have been renamed in accordance with PLOS ONE’s file naming conventions.

2. Please note that PLOS One has specific guidelines on code sharing for submissions in which author-generated code underpins the findings in the manuscript. In these cases, we expect all author-generated code to be made available without restrictions upon publication of the work. Please review our guidelines at <https://journals.plos.org/plosone/s/materials-and-software-sharing#loc-sharing-code> and ensure that your code is shared in a way that follows best practice and facilitates reproducibility and reuse.

**Response:**

We thank the editor for directing us to PLOS ONE’s guidelines for submissions. The datasets used in this study are publicly available and are cited in the manuscript’s Data Availability Statement. MELD, IEMOCAP, and DailyDialog are openly accessible through the repositories listed in the manuscript. No ethical or legal restrictions apply to the sharing of these datasets, and no personally identifiable information is included. The Data Availability Statement in the manuscript specifies the location of each dataset and confirms that all data underlying the findings are fully available without restriction.

3. Your ethics statement should only appear in the Methods section of your manuscript. If your ethics statement is written in any section besides the Methods, please move it to the Methods section and delete it from any other section. Please ensure that your ethics statement is included in your manuscript, as the ethics statement entered into the online submission form will not be published alongside your manuscript.

**Response:**

We thank the editor for this clarification. The ethics statement has been removed from the bottom of the manuscript. This study used publicly available, anonymized datasets: MELD, IEMOCAP, and DailyDialog. We confirm that no ethical constraints are applicable to this work. The study does not involve human subjects, animal experimentation, or sensitive personal data. All data were anonymized prior to analysis and presentation. All datasets were obtained from open repositories and contain no personally identifiable information.

4. Please upload a new copy of Figures 4 - 6 as the detail is not clear. Please follow the link for more information:  <https://journals.plos.org/plosone/s/figures>

**Response:**

We thank the editor for this note. We have rerun the code and redeveloped Figures 4, 5, and 6 with improved resolution, clarity, and legibility. The new figures have been added to the manuscript in accordance with PLOS ONE's figure guidelines. All images are also attached in zip folders with standardised format with tif extensions.

5. We note that Figure 1 in your submission contain copyrighted images. All PLOS content is published under the Creative Commons Attribution License (CC BY 4.0), which means that the manuscript, images, and Supporting Information files will be freely available online, and any third party is permitted to access, download, copy, distribute, and use these materials in any way, even commercially, with proper attribution. For more information, see our copyright guidelines: <http://journals.plos.org/plosone/s/licenses-and-copyright>.

We require you to either (1) present written permission from the copyright holder to publish these figures specifically under the CC BY 4.0 license, or (2) remove the figures from your submission:

1. You may seek permission from the original copyright holder of Figure(s) [#] to publish the content specifically under the CC BY 4.0 license.

We recommend that you contact the original copyright holder with the Content Permission Form (<http://journals.plos.org/plosone/s/file?id=7c09/content-permission-form.pdf>) and the following text:

“I request permission for the open-access journal PLOS ONE to publish XXX under the Creative Commons Attribution License (CCAL) CC BY 4.0 (<http://creativecommons.org/licenses/by/4.0/>). Please be aware that this license allows unrestricted use and distribution, even commercially, by third parties. Please reply and provide explicit written permission to publish XXX under a CC BY license and complete the attached form.”

Please upload the completed Content Permission Form or other proof of granted permissions as an "Other" file with your submission.

In the figure caption of the copyrighted figure, please include the following text: “Reprinted from [ref] under a CC BY license, with permission from [name of publisher], original copyright [original copyright year].”

2. If you are unable to obtain permission from the original copyright holder to publish these figures under the CC BY 4.0 license or if the copyright holder’s requirements are incompatible with the CC BY 4.0 license, please either i) remove the figure or ii) supply a replacement figure that complies with the CC BY 4.0 license. Please check copyright information on all replacement figures and update the figure caption with source information. If applicable, please specify in the figure caption text when a figure is similar but not identical to the original image and is therefore for illustrative purposes only.

**Response:**

We thank the editor for raising this issue. Figure 1 is not taken from any third-party source. It is an original illustration created by one of the co-authors, Amit Pimpalkar, specifically for this manuscript. The dialogue, facial expression representations, emotion labels, and sentiment annotations in the figure were all designed by the author and do not derive from any copyrighted image, dataset, or publication. The names originally shown in the figure were family names of the manuscript co-author, and no permission is therefore required from any other copyright holder.

To address the anonymity requirement during review and to avoid any appearance of using identifiable personal names, we have revised Figure 1 by removing the proper nouns **“Amit”** and **“Swati”** and replacing them with the initials **“A”** and **“S.”** The dialogue content, emotion labels, and sentiment annotations remain unchanged. Since the figure is co-author-created and contains no third-party copyrighted material, the Content Permission Form is not applicable.

We have also updated the figure caption to state the attribution of the figure explicitly and to note that it is for illustrative purposes only. The revised caption reads:

**Figure 1. An example of emotion and sentiment in dialogue.** Original illustration created by the author. The dialogue, emotion labels, and sentiment annotations are author-designed for illustrative purposes only.

We have uploaded the revised figure with the anonymized initials as part of the revised manuscript files.

6. If the reviewer comments include a recommendation to cite specific previously published works, please review and evaluate these publications to determine whether they are relevant and should be cited. There is no requirement to cite these works unless the editor has indicated otherwise.

**Response:**

We thank the editor for this guidance. We have reviewed the publication suggested by the reviewer, Demotte et al., “Enhanced sentiment extraction architecture for social media content analysis using capsule networks,” Multimedia Tools and Applications, 2023.

We evaluated its relevance to the present work and determined that it could be relevant to our discussion of deep learning-based sentiment analysis techniques. The paper introduces a capsule network architecture for social media sentiment analysis that captures hierarchical relationships between words and phrases, which is a useful comparison point for our own sentiment encoding stage. We have therefore cited it as reference [57] in the revised manuscript, within the Related Work section, where we discuss capsule networks alongside other NLP-based sentiment analysis approaches and articulate their limitations for multi-turn dialogue.

**Reviewer's Responses to Questions**

We appreciate that Reviewer #2 found the work technically sound while Reviewer #1 identified partial concerns. We have treated the assessment as constructive and have strengthened the manuscript in the following ways.

- We added a **formal problem statement (Section 1.1)** that defines the four specific gaps addressed and the exact inputs, outputs, and objective, replacing the previously generalized framing. We revised the **Research Gaps and** **contributions (Section 2.5)** so each of the five framework components states its technical novelty and the gap it closes.
- We expanded the **Datasets subsection (Section 4.1)** to report exact train, validation, and test counts for MELD, IEMOCAP, and DailyDialog. We added an **Emotion Clustering Evaluation Protocol (Section 4.2.1)** specifying utterance-level clustering targets, the elbow/silhouette/gap-statistic procedure for selecting the number of clusters, the clustering algorithm, and the evaluation metrics.
- We added a **Computational Complexity and Resource Utilization section (Section 4.3)** with four subsections: **4.3.1 Theoretical Complexity Analysis**, **4.3.2 Empirical Resource Utilization**, **4.3.3 Scalability and Optimization Strategies**, and **4.3.4 Practical Applications and Deployment Considerations**.
- We added a **parameter-matched ablation study (Table 10)** that includes a capacity-matched RoBERTa + classifier baseline at 248.2 M parameters, which achieves only 70.1% F1 versus 78.8% for the full model, demonstrating that the reported gains arise from architectural design rather than increased model capacity.
- We added a **worked DTW alignment example in S1 Appendix** that explicitly walks through Equations 9 through 14, resolving the operational details that were previously only mathematically stated. We added a **detailed ECTSM implementation subsection in S1 Appendix** specifying the point cloud construction, PCA projection, filtration parameters, persistence computation, Deep Set architecture, and training procedure.
- We added **quantified computational limits (Section 4.3.6)** reporting measured persistent homology and Wasserstein timings across point cloud sizes from 20 to 500, replacing the earlier qualitative discussion.
- Finally, we added a **Limitations and Validity of Experimental Results subsection (Section 5)** stating the conditions under which the results hold, including dependence on speaker-turn annotation, the untestable backdoor assumption in the causal module, reward-design sensitivity in the RL component, annotator-pool limits in the human evaluation, and the English-only scope. All conclusions are now stated within these validity boundaries.

**Summary of Revision made**

| **Assessment question** | **Reviewer #1** | **Reviewer #2** | **Revision made** |
| --- | --- | --- | --- |
| 1. Technical soundness | Partly | Yes | Section 1.1 formal problem statement;  Section 2.5 revised contributions; Section 4.1 expanded Datasets, Table 2;  Section 4.2.1 Emotion Clustering Evaluation Protocol;  Section 4.3 Computational Complexity and Resource Utilization (4.3.1 Theoretical Complexity, 4.3.2 Empirical Resource Utilization, 4.3.3 Scalability and Optimization, 4.3.4 Practical Applications);  Table 10 parameter-matched ablation;  S1 Appendix DTW worked example (Eqs. 9–14);  S1 Appendix ECTSM implementation details;  Section 4.3.6 quantified computational limits;  Section 5 limitations and validity; |
| 2. Statistical analysis | N/A | Yes | Five-seed means ± SD explicit; per-run data to be provided as in Tables wherever applicable; |
| 3. Data availability | No | Yes | Data Availability Statement clarified; third-party public datasets specified; |
| 4. Language and clarity | No | Yes | Full proofreading, terminology standardisation, sentence restructuring, Microsoft Word Editor tool to identify and correct grammatical inconsistencies and awkward sentence constructions; |

We thank both reviewers for their time and believe the revised manuscript addresses every concern raised.

**5. Review Comments to the Author**

**Reviewer #1:**

1. It would be better to describe the dataset, with the number of records for training/ testing/ validation. (other than the percentage values)

**Response:**

We thank the reviewer for this helpful comment. We agree that reporting exact split sizes is essential for reproducibility. In the revised manuscript, we have expanded Table 2 to include the number of utterances for training, validation, and test splits for each dataset. The revised table and accompanying text now appear in Section 4, “Datasets.”

**Manuscript revision**

**Section:** Section 4, Experimental design and result analysis, under “Datasets.”

**Action:** We have replaced Table 2 with an expanded version that includes exact counts.

2. What is the computational complexity and the resource utilization of the proposed model. State latency, throughput, memory, or real-time benchmark.

**Response:**

We thank the reviewer for raising this important point. We agree that computational complexity and resource utilization are critical for assessing practical viability. In the revised manuscript, we have added a new subsection, “Computational Complexity and Resource Utilization,” in Section 4. This subsection provides: (i) theoretical complexity for each stage, (ii) empirical measurements of training and inference resource usage, (iii) latency and throughput benchmarks, and (iv) a discussion of real-time feasibility and scalability. The new material is supported by a complexity table and a resource utilization table.

**Manuscript revision**

**Section:** Section 4, Experimental design and result analysis.

**Action:** **Added New subsection 4.3 Computational Complexity and Resource Utilization.**
also discusses the complexity analysis, empirical resource measurements, and real-time benchmark results.

3. What the are possible applications of this proposed model, considering the required resources as well.

**Response:**

We thank the reviewer for this forward-looking comment. We agree that practical applications should be discussed in light of resource requirements. In the revised manuscript, we have added a new subsection 4.3.4, “Practical Applications and Deployment Considerations,” in Section 4. This subsection maps potential applications to their resource needs, deployment feasibility, and ethical considerations. It discusses cloud versus edge deployment, latency and real-time factor constraints, and the need for human oversight in safety-critical domains. The revised text is provided below and now appears in Section 4.3.4.

**Manuscript revision**

**Section:** Section 4, Experimental design and result analysis.

**Action:** **Added New subsection 4.3.4 Practical Applications and Deployment Considerations.**

4. It would be better to extend related studies with techniques on sentiment analysis with NLP techniques such as in <https://doi.org/10.1007/s11042-021-11471-1> and discuss the existing limitations.

**Response:**

We thank the reviewer for pointing us to this relevant work. The referenced study by Demotte et al. proposes an enhanced sentiment extraction architecture for social media content analysis using capsule networks, achieving 86.87% accuracy on the Twitter Sentiment Gold dataset and 82.04% on the CrowdFlower US Airline dataset without relying on linguistic resources. This work represents an important line of research in deep learning-based sentiment analysis, particularly for short, noisy, social media text. We have extended the Related Work section to discuss capsule network approaches alongside other NLP-based sentiment analysis techniques, and we have articulated the specific limitations that motivate our proposed framework.

**Manuscript revision**

**Section:** Section 2, Related Work. Added a new discussion paragraph at the end of Section 2.1.

[57] Demotte, P., Wijegunarathna, K., Meedeniya, D. et al. Enhanced sentiment extraction architecture for social media content analysis using capsule networks. Multimed Tools Appl 82, 8665–8690 (2023). https://doi.org/10.1007/s11042-021-11471-1

5. Extend the ablation study to check whether the modules provide complementary information or whether improvements result primarily from increased model capacity. Try providing results for possible experiments such as parameter-matched ablations; a simple RoBERTa + classifier baseline; RoBERTa + temporal attention; RoBERTa + temporal attention + DTW; topology without DTW; memory without topology; causal module without RL; RL without causal features; full model.

**Response:**

We thank the reviewer for this rigorous and insightful comment. To address this, we have conducted an extensive set of parameter-matched ablations and incremental component experiments. The new results demonstrate that: (1) the proposed modules provide complementary information rather than merely increasing capacity, (2) each stage contributes incrementally to performance, and (3) the full model outperforms parameter-matched baselines that lack specific components. The revised ablation study is presented in a revised Table 10 in Section 4, with accompanying analysis.

**Manuscript revision**

**Section:** Section 4, Ablation study. Replaced the existing Table 8 with an expanded Table 10 that includes parameter-matched baselines and incremental component experiments.

6. It is better if you can provide an example of DTW alignment and quantify the computational cost. The current mathematical description does not fully resolve the operational details.

**Response:**

We thank the reviewer for this practical and important request. We agree that a concrete example of DTW alignment and a clear quantification of its computational cost would improve the operational clarity of the manuscript. In the revised manuscript, the detailed worked example and computational cost analysis have been added to **S1** **Appendix**. The main text in Section 3.1.3 now contains a concise summary. A fully worked example of DTW alignment, including the local and cumulative cost matrices, the optimal alignment path, and a computational cost comparison of exact DTW and FastDTW, is provided in **S1** **Appendix**.

**Manuscript revision**

**Section:** A worked example and computational cost analysis after the existing mathematical description is added to **S1** **Appendix**.

7. Elaborate the implementation details of ECTSM.

**Response:**

We thank the reviewer for this request. We agree that the original manuscript described the mathematical foundations of ECTSM but may not provide sufficient implementation details for reproducibility. In the revised manuscript, we have added in **S1** **Appendix**.. "Implementation Details of ECTSM," which specifies the Vietoris–Rips filtration parameters, the persistent homology computation pipeline, the Deep Set architecture for persistence feature mapping, the training procedure, and all relevant hyperparameters.

**Manuscript revision**

**Section:** The implementation details of ECTSM have been added to **S1** **Appendix**.

8. Discuss situations where the persistent homology can become computationally expensive in high-dimensional datasets.

**Response:**

We thank the reviewer for this important methodological question. We agree that the computational cost of persistent homology in high-dimensional settings deserves explicit discussion. In the revised manuscript, we have added a new subsection 4.3.6, "Quantified Computational Limits of Persistent Homology and Wasserstein Distance," which analyzes the conditions under which persistent homology becomes expensive, the curse of dimensionality, and the mitigation strategies we employ.

**Manuscript revision**

**Section:** Section 4.3, Computational Complexity and Resource Utilization. Added a new subsection 4.3.6.

9. Under emotion-clustering evaluation , state, what clustering targets are used and how the number of clusters is determined.

**Response:**

We thank the reviewer for this important clarification request. We agree that the clustering targets and the method for determining the number of clusters were not sufficiently specified. In the revised manuscript, we have expanded Section 4, Experimental design, to state precisely what is being clustered, how the number of clusters is determined, and how clustering quality is evaluated.

**Manuscript revision**

**Section:** Section 4, Experimental design and result analysis, under "Experimental settings" as 4.2.1 Emotion Clustering Evaluation Protocol.

10. It would be better to quantify the computational limitations associated with persistent homology and Wasserstein calculations, rather than discussed only qualitatively.

**Response:**

We thank the reviewer for this constructive request. We agree that quantitative profiling is more informative than qualitative discussion. In the revised manuscript, we have quantified the computational cost of persistent homology and Wasserstein distance by profiling the existing ECTSM implementation on the MELD dataset using a single A100 GPU and the GUDHI library. We report wall-clock time and peak memory for point cloud sizes $N=20, 50, 100, 200, 500$, corresponding to dialogues of 10 to 250 turns per speaker. The results confirm cubic scaling for persistent homology and super-quadratic scaling for Wasserstein distance. These measurements are now included in subsection 4.3.6, “Quantified Computational Limits of Persistent Homology and Wasserstein Distance,” with a discussion paragraph for Wasserstein distance.

**Manuscript revision**

**Section:** Section 4.3, Computational Complexity and Resource Utilization. Added a new subsection 4.3.6.

11. distinguish cross-dataset benchmark performance from true domain generalization. A held-out domain or out-of-distribution evaluation would provide much stronger evidence.

**Response:**

We thank the reviewer for this important methodological distinction. We agree that our evaluation on MELD, IEMOCAP, and DailyDialog constitutes cross-dataset benchmarking rather than true domain generalization, because all three datasets are English-language, conversation-based, and share similar emotion categories and turn-taking norms. A model performing well on all three has demonstrated robustness across datasets within a narrow domain but not generalization to genuinely different domains such as clinical interviews, non-English dialogues, or social media conversations. We therefore propose a held-out domain protocol for future work: train on the three source datasets and evaluate zero-shot on a target domain such as EmpatheticDialogues or GoEmotions, reporting the performance drop relative to in-domain results to quantify the generalization gap. We believe this transparent discussion appropriately addresses the reviewer’s concern while setting a clear direction for follow-up studies.

**Manuscript revision**

**Section:** Section 5, Conclusion; with following statement

Research can evaluate the framework on a held-out domain, such as EmpatheticDialogues or GoEmotions, to quantify the true domain generalization gap beyond conventional cross-dataset benchmarking.

12. Proofread for language and terminology improvements.

**Response:**

We thank the reviewer for this comment. We have carefully proofread the manuscript and made language and terminology improvements throughout. The revised manuscript has been checked for grammatical accuracy, terminological consistency, and clarity. We have standardized key terms, corrected inconsistencies, and improved sentence structure. A summary of the main corrections is provided below. The full revised manuscript reflects these changes.

**Summary of language and terminology corrections**

| **Issue** | **Original** | **Revised** |
| --- | --- | --- |
| Inconsistent use of "dialog" vs. "dialogue" | Mixed usage | Standardized to "dialogue" throughout |
| Inconsistent use of "sentiment" vs. "emotion" | Sometimes used interchangeably | Clarified: "sentiment" refers to positive/neutral/negative polarity;  "emotion" refers to fine-grained categories such as joy, sadness, anger |
| Inconsistent abbreviation of "dynamic time warping" | Sometimes "DTW", sometimes spelled out | Spelled out at first mention, then "DTW" consistently |
| Inconsistent abbreviation of "Adjusted Rand Index" | Sometimes "ARI", sometimes spelled out | Spelled out at first mention, then "ARI" consistently |
| "Adaptive Causal Decision-Graph Transformer" | Hyphenation inconsistent | Standardized to "Adaptive Causal Decision-Graph Transformer (ACDGT)" |
| "Emotion-Driven Reinforcement Policy Synthesizer" | Hyphenation inconsistent | Standardized to "Emotion-Driven Reinforcement Policy Synthesizer (EDRPS)" |
| "Real-time factor" | Sometimes "RTF", sometimes spelled out | Defined at first use, then "RTF" consistently |
| "Wasserstein distance" | Sometimes "Wasserstein metric" | Standardized to "Wasserstein distance" |
| "Vietoris–Rips" | Sometimes "Vietoris–Rips" with hyphen | Standardized to dash "Vietoris-Rips" |
| Geometry Understanding in Higher Dimensions "GUDHI" | Sometimes "Gudhi" | Standardized to "GUDHI" |
| "PPO" | Sometimes "proximal policy optimization" without abbreviation | Defined at first use, then "PPO" consistently |
| standard deviation (SD) | Sometimes "standard deviation (SD) " without abbreviation | Defined at first use, then "SD" consistently |
| "Parameter matched" | Sometimes "parameter- matched" | Standardized to "parameter matched" |
| "Real-world" | Sometimes "real world" | Standardized to "real-world" when used as adjective |
| Sentence-level grammar | Several run-on sentences and comma splices | Corrected for clarity and concision |
| Article usage | Occasional missing or incorrect articles | Corrected throughout |
| Subject-verb agreement | Occasional mismatches | Corrected throughout |
| Tense consistency | Mixed past and present tense in methodology | Standardized to present tense for methodology, past tense for experiments |
| Overly long sentences | Several sentences exceeding 30 words | Split into shorter sentences |
| Numerical formatting | Inconsistent use of "percent" vs. "%" | Standardized to "%" in tables, "percent" in text |
| Ambiguous pronoun | “This is a limitation for any coherent existence of emotional understanding” | “This limitation prevents coherent emotional understanding in long dialogues” |
| Generalized claim | “Dialogue systems must improve upon natural language processing….” | Dialogue systems must advance beyond conventional Natural Language Processing (NLP) by encoding speaker identity and temporal order capabilities that standard sentence-level NLP does not provide while also striving to understand, adapt to, and generate emotional expressions. |
| Vague phrasing | “Emotions become the central objects for dialog work” | “Emotion representations become the input to downstream policy decisions” |
| Run-on sentence | “Current models do not provide such a mechanism to differentiate between emotional triggers and incidental emotional expressions, which hinders their application in decision-making tasks that involve emotional causality understanding” | “Current models do not distinguish emotional triggers from incidental expressions. This limits their use in decision-making tasks that require causal understanding of emotion.” |

We believe these corrections improve the readability, professionalism, and terminological precision of the manuscript. We thank the reviewer for prompting this thorough proofreading.

**Reviewer #2:**

1. The manuscript should be proofread further, to improve the presentation clarity. (The intended meaning often lacks clarity)

**Response:**

We thank the reviewer for this comment. We agree that the presentation clarity required improvement. We have proofread the manuscript thoroughly and corrected grammar, sentence structure, terminology consistency, and ambiguous phrasing. We have also split overly long sentences, replaced vague pronouns with explicit nouns, and standardized technical terms throughout. A summary of the main corrections is provided below, and the full revised manuscript reflects these changes.

**Summary of language and terminology corrections**

| **Issue** | **Original** | **Revised** |
| --- | --- | --- |
| Inconsistent use of "dialog" vs. "dialogue" | Mixed usage | Standardized to "dialogue" throughout |
| Inconsistent use of "sentiment" vs. "emotion" | Sometimes used interchangeably | Clarified: "sentiment" refers to positive/neutral/negative polarity;  "emotion" refers to fine-grained categories such as joy, sadness, anger |
| Inconsistent abbreviation of "dynamic time warping" | Sometimes "DTW", sometimes spelled out | Spelled out at first mention, then "DTW" consistently |
| Inconsistent abbreviation of "Adjusted Rand Index" | Sometimes "ARI", sometimes spelled out | Spelled out at first mention, then "ARI" consistently |
| "Adaptive Causal Decision-Graph Transformer" | Hyphenation inconsistent | Standardized to "Adaptive Causal Decision-Graph Transformer (ACDGT)" |
| "Emotion-Driven Reinforcement Policy Synthesizer" | Hyphenation inconsistent | Standardized to "Emotion-Driven Reinforcement Policy Synthesizer (EDRPS)" |
| "Real-time factor" | Sometimes "RTF", sometimes spelled out | Defined at first use, then "RTF" consistently |
| "Wasserstein distance" | Sometimes "Wasserstein metric" | Standardized to "Wasserstein distance" |
| "Vietoris–Rips" | Sometimes "Vietoris–Rips" with hyphen | Standardized to dash "Vietoris-Rips" |
| Geometry Understanding in Higher Dimensions "GUDHI" | Sometimes "Gudhi" | Standardized to "GUDHI" |
| "PPO" | Sometimes "proximal policy optimization" without abbreviation | Defined at first use, then "PPO" consistently |
| standard deviation (SD) | Sometimes "standard deviation (SD) " without abbreviation | Defined at first use, then "SD" consistently |
| "Parameter matched" | Sometimes "parameter- matched" | Standardized to "parameter matched" |
| "Real-world" | Sometimes "real world" | Standardized to "real-world" when used as adjective |
| Sentence-level grammar | Several run-on sentences and comma splices | Corrected for clarity and concision |
| Article usage | Occasional missing or incorrect articles | Corrected throughout |
| Subject-verb agreement | Occasional mismatches | Corrected throughout |
| Tense consistency | Mixed past and present tense in methodology | Standardized to present tense for methodology, past tense for experiments |
| Overly long sentences | Several sentences exceeding 30 words | Split into shorter sentences |
| Numerical formatting | Inconsistent use of "percent" vs. "%" | Standardized to "%" in tables, "percent" in text |
| Ambiguous pronoun | “This is a limitation for any coherent existence of emotional understanding” | “This limitation prevents coherent emotional understanding in long dialogues” |
| Generalized claim | “Dialogue systems must improve upon natural language processing….” | Dialogue systems must advance beyond conventional Natural Language Processing (NLP) by encoding speaker identity and temporal order capabilities that standard sentence-level NLP does not provide while also striving to understand, adapt to, and generate emotional expressions. |
| Vague phrasing | “Emotions become the central objects for dialog work” | “Emotion representations become the input to downstream policy decisions” |
| Run-on sentence | “Current models do not provide such a mechanism to differentiate between emotional triggers and incidental emotional expressions, which hinders their application in decision-making tasks that involve emotional causality understanding” | “Current models do not distinguish emotional triggers from incidental expressions. This limits their use in decision-making tasks that require causal understanding of emotion.” |

2. The proposed solution is briefly described. A lot of information should be given here about the consideration of the developed architecture.

**Response:**

We thank the reviewer for this constructive comment. We agree that the main text provides only a high-level overview of the five-stage architecture and that additional detail on the design rationale, component interactions, and implementation considerations would improve clarity and reproducibility. In the revised manuscript, we have added an **S1** **Appendix** that expands on the motivation for each stage, the design choices made, the interactions between modules, and the rationale for key hyperparameters.

**Manuscript revision**

**Section:** A worked example and computational cost analysis after the existing mathematical description is added to **S1** **Appendix**.

**Section:** The implementation details of ECTSM are added to **S1** **Appendix**.

3. How do you deal with dataset imbalance problems? Did you consider any data augmentation techniques?

**Response:**

We thank the reviewer for this important question. All three datasets are imbalanced, with neutral and joy dominating. We addressed this using inverse-frequency class weighting in the training loss; focal loss was evaluated but produced higher variance. We did not apply data augmentation because emotional semantics are sensitive to lexical edits, and generative augmentation risks distribution shift. We have revised Section 4.1 to explicitly detail our approach to imbalance handling and to identify emotion-conditioned generation as a promising direction for future work. Table 2 has been expanded to report exact train, validation, and test sizes.

**Manuscript revision**

**Section:** Section 4, Experimental design and result analysis, under “Datasets.”
**Action:** We have replaced Table 2 with an expanded version that includes exact counts.

4. Discuss the limits of the proposed approach and the validity of experimental results.

**Response:**

We thank the reviewer for this important request. We agree that the original manuscript did not sufficiently discuss the limitations of the proposed approach or the validity boundaries of the experimental results. In the revised manuscript, we have added a new subsection 4.3.6, "Quantified Computational Limits of Persistent Homology and Wasserstein Distance," which analyzes the conditions under which persistent homology becomes expensive, the curse of dimensionality, and the mitigation strategies we employ. In the revised manuscript, we have revised Section 5, which explicitly states the conditions under which the results hold and the conditions under which they may not generalize.

**Manuscript revision**

**Section:** Section 4.3, Computational Complexity and Resource Utilization. Added a new subsection 4.3.6.

**Section:** Section 5, Conclusion. Revised the current content.

5. There are generalized statements and hence author needs to identify precisely the problem they wish to solve.

**Response:**

We thank the reviewer for this important observation. We agree that the Introduction contained broad, generalized statements about emotionally intelligent dialogue systems without precisely stating the problem being solved. In the revised manuscript, we have added a new subsection 1.1, “Problem Statement,” which defines the problem formally and lists the four specific gaps the work addresses. We have also revised the opening paragraphs of the Introduction to remove generalized claims and replace them with specific, measurable statements. The five stages of the proposed framework correspond directly to these requirements: CMLSE preserves speaker and temporal structure, ECTSM captures topological emotion configurations, EMR-GAN maintains long-range emotional memory, ACDGT estimates causal influence, and EDRPS generates the policy. This problem statement replaces the generalized framing in earlier drafts and defines the precise scope of the contribution.

**Manuscript revision**

**Section:** Section 1, Introduction. Added subsection 1.1 after the current introductory paragraphs.

6. Results need to be briefly discussed.

**Response:**

We thank the reviewer for this comment. We agree that the original Results section presented numerical outcomes without sufficient interpretation. In the revised manuscript, we have added new subsections and a dedicated discussion paragraph in Section 4 specifically, 4.1 (Datasets), 4.2.1 (Emotion Clustering Evaluation Protocol), and 4.3 (Computational Complexity and Resource Utilization), along with expanded observation accompanying in Tables 2, 8, 9 and 10. Each of these additions explicitly states what the result means, why the proposed model outperforms the baselines, and what the practical implications are for deployment and future work.

**Manuscript revision**

**Section:** Section 4, after Tables 2, 8, 9, and 10. Add a brief discussion paragraph after each.

7. Overall, The author must highlight the scientific contribution, describe better his/her approach, and give enough information to compare the results.

**Response:**

We thank the reviewer for this comment. We note that the scientific contributions are stated at the end of Section 2, in the paragraph beginning “To address these gaps, this work proposes a novel five-stage architecture,” which lists the five framework contributions. We agree, however, that this list was not sufficiently emphasized and that the approach description and comparison protocol needed strengthening.

In the revised manuscript, we have therefore made targeted changes rather than duplicating the contribution list. First, we have sharpened the existing contribution paragraph in Section 2 so that each of the five contributions states the specific technical novelty and the gap it closes, rather than describing the component generically. Second, we have added a detailed architecture and results in appendix (**S1 Appendix**) that explains the design rationale, component interactions, and implementation considerations for each stage, so that the approach is described operationally rather than only mathematically. Third, we have added a comparison protocol paragraph in Section 4 that specifies the baselines, training conditions, tuning budget, splits, tokenizer, sequence length, early stopping criterion, and random seeds, so that readers have enough information to compare results fairly.

**Manuscript revision**

**Section 2, end of Section 2.5 (Research Gaps and Contributions):** Rewriten the five-point contribution list so each point states novelty and gap explicitly.

We also used the Microsoft Word Editor tool to identify and correct grammatical inconsistencies and awkward sentence constructions. After revision, the manuscript achieved a 100% Editor Score. All corrections have been incorporated into the revised version of the manuscript.

The entire manuscript has been copy-edited wherever needed. Major changes include removal of redundant phrases, images/table discussion, correction of subject-verb agreement, and improvement of technical terminology. These changes improve the manuscript’s readability, reduce redundancy, and ensure a clearer presentation of the proposed framework.

The authors are also grateful to all the reviewers and Editor who have contributed through their comments and constructive recommendations which have made significant impact in making the manuscript clear, deep and overall, of good quality. The revisions that we made based on the inputs of all the reviewers and Editor, now feel that the manuscript is in an improved and presentable state to be accepted and published. The reviewers and Editor have indeed helped us to work on this work, and we really appreciate the time and effort put into it.

Sincerely,
Corresponding Author

(On the behalf of all authors)
